# Combined associations of physical activity, diet quality and their trajectories with incidence of diabetes and cardiovascular diseases in the EPIC-Norfolk Study

**DOI:** 10.1038/s41598-025-93679-x

**Published:** 2025-04-16

**Authors:** Shayan Aryannezhad, Fumiaki Imamura, Alexander Mok, Nicholas J. Wareham, Nita G. Forouhi, Soren Brage

**Affiliations:** 1 MRC Epidemiology Unit, Institute of Metabolic Science, University of Cambridge School of Clinical Medicine, Cambridge Biomedical Campus, Cambridge, CB2 0QQ UK; 2 Brenner Centre for Molecular Medicine, Singapore Institute for Clinical Sciences, Agency for Science, Technology and Research (A*STAR), Medical Drive, Singapore, 117609 Republic of Singapore

**Keywords:** Physical activity, Energy expenditure, Mediterranean diet, Trajectory, Cardiovascular disease, Diabetes mellitus, Cardiovascular diseases, Diabetes, Epidemiology

## Abstract

The combined impact of physical activity (PA), diet quality, and their change over time on cardiometabolic disease risk remains uncertain. We aimed to examine the associations between the combined trajectories of these health behaviours and the incidence of diabetes mellitus (DM) and cardiovascular diseases (CVD) among 9,276 middle-aged adults. In the EPIC-Norfolk study, longitudinal changes in PA energy expenditure (∆PAEE) and Mediterranean diet score (∆MDS) were derived from repeated measurements between 1993 and 2004. Over a median follow-up of 18 years, 968 participants developed DM and 2,540 developed CVD by 2022. In mutually adjusted Cox regression models, HR (95%CI) per 1-SD higher ∆PAEE and ∆MDS were 0.87 (0.81–0.94) and 0.92 (0.86–0.99) for incident DM, and 0.94 (0.89–0.99) and 0.93 (0.88–0.97) for incident CVD, respectively. Compared to participants with sustained low PAEE and MDS, those with sustained higher levels of both traits had a 40% (21-55%) lower incidence of DM and a 25% (11-37%) lower CVD incidence. At the population level, consistent favourable levels of activity and diet quality could reduce cumulative incidence of DM by 22% and CVD by 16%. The greatest benefit arises when an active lifestyle is combined with a high-quality diet, initiated earlier in adulthood, and maintained over time.

**Supplementary Information:**

The online version contains supplementary material available at 10.1038/s41598-025-93679-x.

## Introduction

The spectrum of cardiometabolic diseases (CMD) encompasses metabolic disorders, including diabetes mellitus (DM) and cardiovascular diseases (CVD). Both DM and CVD contribute to global disability and mortality, placing a substantial burden on healthcare systems. The Global Burden of Disease study estimated that 537 million people worldwide had DM in 2021, and this number was predicted to rise to 1.3 billion by 2050^1^. Similarly, the rising trend of CVD, particularly ischemic heart disease (IHD) and stroke, stands as a substantial challenge to public health. Being responsible for more than 13 million deaths globally in 2021, IHD and stroke are the world’s predominant causes of mortality^2^.

Physical inactivity and poor diet quality are the leading modifiable risk factors of CMD, based on recent meta-analyses of observational studies^3–6^. Evidence from randomised controlled trials indicates that increasing physical activity (PA) and improving diet quality in combination reduces the risk of incident DM and incident CVD, and this effect is greater compared with a diet-alone or a activity-alone intervention^7,8^. However, the knowledge derived from such trials is limited in generalisability because of their generally short follow-up durations (active intervention lasting typically a few months), small sample sizes (usually involving up to a few hundred participants) and including predominantly individuals with pre-existing cardiometabolic risk factors. As long-term maintenance of health behaviour modifications introduced in intervention studies is often not achieved^9^, to reach conclusions for wide application, complimentary evidence can be sought from large observational cohort studies covering longer periods of the life course and with repeat measures of health behaviours over time.

A few longitudinal prospective studies have shown that increasing PA over time or maintaining consistent high levels of PA is linked to a lower risk of developing CMD. These studies, however, frequently overlook the role of diet quality as a shared exposure or even as a potential confounder in this relationship^10–20^. On the other hand, improving diet quality throughout life is associated with lower incidence of CMD^21–27^. However, there is a lack of longitudinal data concerning the joint impact of PA and diet quality on those cardiometabolic outcomes. As the development of CMD may reflect the complex interplay of various behavioural factors^28^, it is essential to consider the combined and concurrent association of PA and diet quality trajectories in this context to better understand their dynamic nature and explore possible synergistic or compensatory effects between them.

The aim of this study was to evaluate the separate and combined associations of physical activity and diet quality, at baseline and within-person changes over time, with the incidence of DM and CVD in a population-based cohort of adults residing in the UK.

## Methods

### Study population

Within the framework of the European Prospective Investigation of Cancer (EPIC), the EPIC-Norfolk study recruited 25,639 men and women aged 40 to 79 years resident in Norfolk, UK at the baseline health check from 1993 to 1997 (HC1). This population-based cohort study then further collected data prospectively through in-person visits at clinics and postal questionnaires. Details of the study design have been previously described^29^. In this current study, we evaluated available data of habitual diet collected at both HC1 and the follow-up, HC2 (1998–2000) and of PA collected at HC1 and a postal follow-up (2002–2004). Those with prevalent DM during the assessment period (1993–2004) were excluded from the analysis of incident DM, and similarly those with prevalent CVD during the same period were excluded from the analysis of incident CVD. Figure S1 shows the flow diagram of the analytical sample including at least 8,800 participants. Of those, 5,493 participants underwent further PA and diet assessment at HC3 (2005–2011), enabling our secondary analysis of repeated changes assessed at the same time (HC1 to HC3 for both PA and diet).

### Assessment of PA and diet

Physical activity was assessed using the short-EPIC physical activity questionnaire, a validated instrument originally developed for the EPIC study and later adapted for use in the EPIC-Norfolk study^29^. The questionnaire asked about habitual PA over the past year collected information on occupational and leisure-time PA. Occupational PA was categorised as either unemployed/ sedentary work, standing, or physical / heavy manual work. Leisure-time PA averaged summer and winter duration of cycling and physical exercise and was collapsed into four categories. These two domains of PA were then combined, validated and calibrated against individually calibrated heart rate monitoring and combined movement and heart rate monitoring to derive physical activity energy expenditure (PAEE, in units of kJ/day/kg), as previously described^30–32^.

A 130-item semi-quantitative food frequency questionnaire (FFQ) that was originally developed in the early 1990s and described previously was implemented in the EPIC-Norfolk study to assess participants’ habitual diet over the past year^33^. The validity of this FFQ for major foods and nutrients was assessed previously in a sub-sample of EPIC-Norfolk, against 16-day weighed dietary records, 24-hour dietary recall, and specific biomarkers^34–36^. The Mediterranean diet score (MDS) was used to assess overall diet quality, previously developed as a measure of dietary adherence to the Mediterranean dietary pyramid^37,38^. In brief, the MDS was a continuous score from 0 to 15 points based on how much reported dietary consumption was consistent with the recommendation of the dietary pyramid, based on 15 dietary components: vegetables, legumes, fruits, nuts, cereals, dairy, fish, red meat, processed meat, white meat, eggs, potatoes, sweets, alcohol and olive oil.

### Assessment of covariates

Baseline assessment of covariates was carried out at HC1 (1993-97) and repeated at HC2 (1998–2000), postal follow-up (2002–2004) and HC3 (2005–2011, sensitivity analysis). At the clinical assessments, trained research nurses obtained anthropometric measures using standardised protocols from which body mass index (BMI) in kg/m^2^ and waist circumference (WC) in cm were computed^39^. Systolic and diastolic blood pressure (SBP and DBP) was assessed while seated. Serum triglycerides (TG), low-density lipoprotein cholesterol (LDL-C), and high-density lipoprotein cholesterol (HDL-C) were measured in non-fasted blood samples^39^. Participants self-reported their age, sex, marital status, smoking status, education level and employment status. For this study, smoking status was categorised into three levels (current smokers; former smokers; never smokers); education level was categorised into two levels (General Certificate of Education [GCE] Ordinary Level or below; GCE Advanced Level, bachelor’s degree, and above) and employment type was categorised into three levels (unemployed to semi-skilled work; skilled workers; managers and professionals). Medication use, comorbidities, and family history of DM and CVD were self-reported. Additionally, data from hospital episode statistics were used to ascertain updated medical histories on prevalent diseases for the entire exposure assessment period (1993–2004).

### Outcome ascertainment

In this study, we defined at-risk periods as the last assessment of primary exposures (changes in PAEE and MDS), i.e. from the date of returning the last postal follow-up questionnaire in 2002-04 until 2020 for DM and until 2022 for CVD. Incident DM was ascertained if participants self-reported both physician’s diagnosis and anti-diabetic medication use or if one or more sources of information was obtained: HbA1c ≥ 48mmol/mol (6.5%), measured at clinics in study follow-ups or by record linkage with Norfolk and Norwich University Hospital which served the population of Norfolk; diabetes present in Hospital Episode Statistics (HES) for in-hospital and outpatient admission records; diabetic eye screening programme; and diabetes noted as cause-of-death in mortality records from the Office of National Statistics. Incident CVD was defined as a composite outcome of incident IHD and stroke; this was ascertained if hospital admission records and mortality records included the international classification of diseases versions 9 and 10 (ICD-9 and ICD-10) codes of 410–414 or I20-I25 for IHD, 430–438 or I60-I69 for incident stroke. Fatal CVD, fatal IHD and fatal stroke were identified based on mortality records, where the cause of death was attributed to the respective diseases.

### Statistical analysis

Longitudinal within-person changes in PAEE (∆PAEE) and MDS (∆MDS) were calculated by subtracting the corresponding exposure value at the baseline (HC1) from the value at the repeated assessment, divided by the elapsed time (in years). These change variables were expressed in units of kJ/kg/day per year for ∆PAEE and score points per year for ∆MDS. For analyses of disease associations, four mutually adjusted exposure variables (baseline PAEE, ∆PAEE, baseline MDS and ∆MDS) were included simultaneously as continuous exposure variables.

Cox proportional hazards regression models were fitted to estimate hazard ratios (HRs) and 95% confidence intervals (95% CI) for the prospective associations between PA, diet and incidence of diseases. Time at risk (follow-up person-years) were calculated from the time of the last repeated questionnaire assessment until the time of incident DM or CVD, death, or the censor date (31st March 2020 for DM analysis and 31st March 2022 for CVD analysis), whichever occurred first. Proportional-hazards assumption was tested using Schoenfeld residuals. Linear relationships between the log-hazard and the continuous covariates were assessed visually by plotting Martingale residuals.

To investigate the specific trajectory combinations of PA and diet over time, we dichotomised both exposures (Low/High) at each of baseline and repeated assessments, creating sixteen different trajectory groups. Specifically, these were assigned according to dichotomous achievement of a healthy PA behaviour (PAEE ≥ 5 kJ/kg/day, corresponding to the World Health Organization minimum physical activity recommendation of 150 min/week of moderate-to-vigorous PA) and a healthy overall diet quality (above median of MDS points in the cohort, corresponding to moderate adherence to the Mediterranean diet^38^). In the Cox proportional hazards regression analysis, the sixteen trajectory groups were entered as categorical variables, keeping the group with low PA and low diet quality at both baseline and repeated assessments (Group 1; G1) as the reference in the joint analysis. We also conducted stratified analyses by the four PA-diet categories at baseline. In each stratum, the reference group was set as the group that maintained the baseline levels of PAEE and MDS over the entire assessment period.

Different multivariable regression models were developed to account for potential confounders and mediators. Model 1 adjusted for sex and age. Model 2 further adjusted for other potential confounders and included socioeconomic variables (education, social class, marital status), family history of DM, family history of myocardial infarction (MI), and time-updated variables for smoking, hormone replacement therapy, total energy intake, lipid-lowering drugs, antihypertensive drugs, anti-diabetes drugs (only when CVD was the outcome), prevalent diseases (prevalent CVD when DM was the outcome, prevalent DM when CVD was the outcome). Model 3 additionally adjusted for time-updated adiposity markers (BMI and WC, entered as baseline values and their change over time [∆]), which may be considered to be either potential confounders, potential mediators, or both, in the association between health behaviours and incident CMD. Model 4 further adjusted for potential mediators and included time-updated variables for blood-based risk factors (TG, LDL, and HDL) and resting blood pressure (SBP and DBP). Missing covariates were imputed using multiple imputation with chained equations. Possible synergistic or compensatory effects between PA, diet quality and their changes over time were examined by performing tests of interaction between these exposures on both multiplicative and additive scales (information in supplementary materials). Stratified analyses were performed to test for potential effect modification by age, sex, BMI, and smoking.

To assess population impact, we compared observed and estimated cumulative incidence under two counterfactual scenarios. In scenario 1, adjusted cumulative incidence was estimated for each of DM and CVD, under the assumption that the whole population had consistently low PAEE and low MDS over the assessment period; in scenario 2, the modelling assumed that the whole population had consistently high PAEE and high MDS.

A series of ancillary analyses were performed to examine if the primary findings were robust to different modelling approaches. The Cox regression models were repeated in complete-case analyses, without imputation of missing covariates. We extended the assessment period up to HC3 (2005–2011) in a sub-sample with available HC3 data; relevant ∆PAEE and ∆MDS variables were re-calculated covering a longer exposure period (and shorter follow-up for incidence of diseases). Additionally, to mitigate the possibility of reverse causation, we excluded individuals who had a diagnosis of DM or CVD (CMD) or died within two years of the last repeated assessment of PA and diet. Fractional polynomial modelling was conducted to examine non-linear associations and visualising dose-response curves. Moreover, to examine potential impact of exposure oversimplification resulting from dichotomising the PAEE and MDS variables, an analysis was conducted using trajectories based on three exposure levels of PA and diet at baseline and repeat assessments, yielding 9 trajectory groups for each health behaviour (controlling for the other). All analyses were performed using Stata software version 16.

### Ethics approval and consent to participate

The study was approved by the Norfolk District Health Authority Ethics Committee (Ref: 98NC01) and adhered to the Declaration of Helsinki. Informed consent was obtained from all participants.

## Results

### Participant characteristics

At the baseline assessment (1993-97) participants had a mean age of 58.3 (SD 8.7) years and 58.8% were women (Table 1). During the exposure assessment period, a decline in PAEE was observed, commencing at 6.04 (SD 4.64) kJ/kg/day, with an annual reduction of 0.13 kJ/kg/day until the repeated assessment in 2002-04 (median assessment period of 7.6 years [IQR 7.1–8.1]). Simultaneously, there was an increase in the MDS, commencing at 8.66 [SD 1.29] points, with a yearly increase of 0.03 points until the repeated assessment in 1998–2000 (median assessment period of 3.6 years [IQR 3.1-4]). Subsequent to the last exposure assessment, a total of 968 participants developed DM (median follow-up of 16.1 years [IQR 11.8–17.1], 123,036 person-years), and a total of 2,540 participants developed CVD (median follow-up of 18.0 years [IQR 11.0-18.9], 129,484 person-years).

**Table 1:** Participant characteristics at baseline and repeated assessments of the EPIC-Norfolk study (n total = 9,276).

Characteristic	Baseline assessment (1993–1997)	Repeated assessment (1998–2004)
Demographics		
Age (years)	58.3 (8.7)	61.4 (8.8)
Women (%)	5,453 (58.8)	
Education: GCSE/O level equivalent or below (%)	3,872 (41.8)	
Occupation: Unemployed to semi-skilled work (%)	1,442 (15.6)	
Occupation: Skilled workers (%)	3,422 (36.9)	
Occupation: Managers & professionals (%)	4,412 (47.5)	
Metabolic risk factors		
Body mass index (kg/m^2^)	25.9 (3.7)	26.5 (3.9)
Waist circumference (cm)	86.3 (11.9)	87.2 (12.3)
Systolic blood pressure (mmHg)	134.4 (17.6)	135.0 (17.8)
Diastolic blood pressure (mmHg)	82.2 (10.9)	82.1 (10.9)
Triglycerides (mmol/L)	1.73 (1.4)	1.84 (1.06)
HDL-Cholesterol (mmol/L)	1.44 (0.44)	1.51 (0.46)
LDL-Cholesterol (mmol/L)	3.94 (1.03)	3.78 (1.04)
Prevalent diseases, medication, and family history		
DM during the assessment period (%)	422 (4.6)	
CVD during the assessment period (%)	476 (5.1)	
Statins (%)	100 (1.1)	353 (3.8)
Anti-hypertensive drugs (%)	1,264 (13.6)	1,894 (20.4)
Anti-diabetic drugs (%)	81 (0.9)	122 (1.3)
Hormone Replacement Therapy (current or former) (% in women)	1,820 (33.4)	2,148 (39.4)
Family history of diabetes mellitus (%)	1,253 (13.5)	
Family history of myocardial infarction (%)	3,448 (37.2)	
Health behaviours		
PAEE (kJ/kg/day)	6.04 (4.64)	5.01 (4.64)^†^
ΔPAEE (kJ/kg/day per year)	-	-0.13 (0.65)^†^
MDS points	8.55 (1.30)	8.66 (1.29)
ΔMDS (points per year)	-	0.03 (0.33)
Current smoker (%)	730 (7.9)	623 (6.7)
Former smoker (%)	3,658 (39.4)	3,774 (40.7)
Energy intake (kcal/day)	2060 (583)	1964 (559)
Alcohol (g/d)	8.5 (12.4)	8.8 (12.5)

Data are presented as mean (SD) or number (%).

HDL = high density lipoprotein; LDL = low density lipoprotein; DM = diabetes mellitus, CVD = cardiovascular diseases; PAEE = physical activity energy expenditure; ΔPAEE = over time changes in PAEE; MDS = Mediterranean diet score; ΔMDS = over time changes in MDS.

The *n* = 9,276 sample consists of individuals with baseline and repeated dietary and physical activity data, who do not have prevalent DM and CVD as a combined condition during the assessment period.

^†^For all variables, except those indicated by a dagger symbol (†), the repeated assessment period is Health Check 2 (HC2: 1998–2000). For the variables indicated by the dagger symbol, the repeated assessment period is the time of Postal Follow-Up (PFU: 2002–2004).

### Associations of baseline and changes in PA and diet quality with incidence of DM and CVD

Table 2 shows HRs with 95% confidence intervals for the associations of mutually adjusted exposures with incident DM and incident CVD. In the model adjusted for potential confounders (Model 2), for each 1-SD higher baseline PAEE (4.64 kJ/kg/day), ∆PAEE (0.65 kJ/kg/day per year), baseline MDS (1.30 points) and ∆MDS (0.33 points per year), the HRs (95% CI) for incident DM were 0.89 [0.82 to 0.96], 0.87 [0.81 to 0.94], 0.89 [0.83 to 0.96] and 0.92 [0.86 to 0.99], respectively. For incident CVD, the HRs and 95% CIs were 0.93 [0.88 to 0.98], 0.94 [0.89 to 0.99], 0.94 [0.90 to 0.99] and 0.93 [0.88 to 0.97], respectively. Associations were generally consistent among subtypes of CVD, with a slightly higher magnitude observed in fatal cases (fatal CVD, fatal IHD, and fatal stroke) compared to incident cases of all CVD, incident IHD, and incident stroke (Table S1). Associations with IHD were slightly stronger for PA whereas associations for stroke were stronger for diet. Adjustment for adiposity markers slightly attenuated the associations for incident DM but not for incident CVD (Model 3).

**Table 2:** Hazard ratios with 95% confidence intervals for the associations of mutually adjusted exposures with incident cardiometabolic diseases in the EPIC-Norfolk study.

Outcome	Exposures^‡^	Model 1 (minimally adjusted)	Model 2 (further adjustment for potential confounders)	Model 3 (further adjustment for adiposity)
Incident DM 968 cases 123,036 person-years	Baseline PAEE	0.87 (0.80 to 0.94)	0.89 (0.82 to 0.96)	0.95 (0.88 to 1.03)
	ΔPAEE	0.85 (0.79 to 0.92)	0.87 (0.81 to 0.94)	0.90 (0.84 to 0.98)
	Baseline MDS	0.85 (0.79 to 0.91)	0.89 (0.83 to 0.96)	0.91 (0.85 to 0.99)
	ΔMDS	0.90 (0.84 to 0.96)	0.92 (0.86 to 0.99)	0.96 (0.89 to 1.03)
Incident CVD 2,540 cases 129,484 person-years	Baseline PAEE	0.91 (0.87 to 0.95)	0.93 (0.88 to 0.98)	0.94 (0.90 to 0.99)
	ΔPAEE	0.92 (0.88 to 0.97)	0.94 (0.89 to 0.99)	0.94 (0.90 to 0.99)
	Baseline MDS	0.93 (0.89 to 0.98)	0.94 (0.90 to 0.99)	0.94 (0.90 to 0.99)
	ΔMDS	0.92 (0.88 to 0.96)	0.93 (0.88 to 0.97)	0.93 (0.89 to 0.97)

HRs per SD difference in each exposure are presented. Sample size *n* = 8,854 for DM outcome (end of follow-up: March 2020) and *n* = 8,800 for CVD outcomes (end of follow-up: March 2022). All coefficients are mutually adjusted for the four primary exposures (baseline PAEE, ΔPAEE, baseline MDS, ΔMDS). Covariates in the Models: Model 1: sex, age. Model 2: variables in Model 1 + education, social class, marital status, FH of DM, FH of MI; and time updated variables for smoking, HRT, total energy intake, lipid-lowering drugs, antihypertensive drugs, anti-diabetes drugs (only when CVD is the outcome), prevalent diseases (prevalent CVD when DM is the outcome, prevalent DM when CVD is the outcome). Model 3: variables in Model 2 + time updated variables for BMI, WC.

^‡^SD increment in baseline PAEE equals to 4.64 kJ/kg/day, in ΔPAEE equals to 0.65 kJ/kg/day per year, in baseline MDS equals to 1.30 points, and in ΔMDS equals to 0.33 points per year.

CI, Confidence interval; CVD, cardiovascular diseases; EPIC, European Prospective Investigation of Cancer and nutrition; HR, hazard ratio; MDS, Mediterranean diet score; PAEE, physical activity energy expenditure; SD, standard deviation.

Further adjustment for potential mediators of CMD in Model 4 yielded minimal differences when compared to the findings in Model 3 (see Table S2). Findings remained consistent when excluding participants with missing covariates (Table S3) and across various other sensitivity analyses, including when exposure assessment period was extended to HC3 (Table S4), when excluding events that occurred within two years of the last measurement (Table S5), and when the dose-response associations with outcomes were assessed using non-linear modelling (Figure S2). To assess potential selection bias, HR (95% CI) for baseline exposures was derived for the entire cohort sample, which was comparable to its equivalent for the main analytical sub-sample with repeat assessments (Table S6).

Interactions between different combinations of the exposures for the association with DM and CVD were tested on both multiplicative and additive scales (Table S7). No significant interactions were found for any combination of exposures on incident DM. However, for CVD, we identified three significant interactions. First, the inverse association between changes in ΔPAEE and CVD risk was stronger in individuals with higher baseline PAEE (Baseline PAEE × ΔPAEE). Second, the inverse association between baseline MDS and CVD risk was stronger in those with higher baseline PAEE (Baseline PAEE × Baseline MDS). Lastly, we observed an interaction between ΔPAEE and ΔMDS, indicating that simultaneous improvements in physical activity and diet quality were associated with a greater reduction in CVD risk than sum of the improvements in either behaviour alone.

The associations between four continuous exposures and the disease endpoints in population subgroups were examined through stratified analyses, considering potential effect modifiers by age, sex, BMI, and smoking status at baseline (Figure S3). Tests for subgroup interaction were non-significant for all stratified analyses (P value ≥ 0.05 in all analyses).

### Joint associations of trajectories of PA and diet quality with incidence of DM

**Fig. 1:**
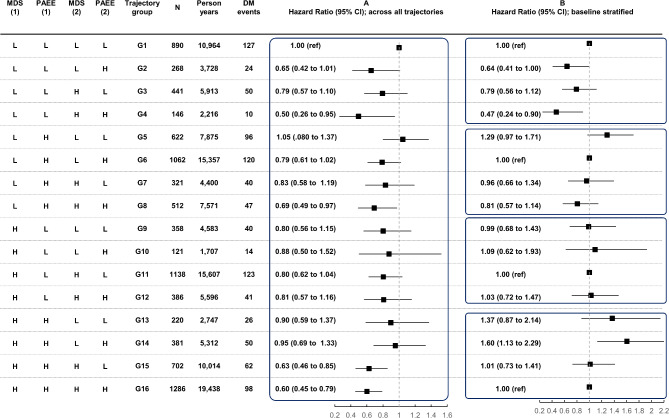
Joint associations of different trajectories of physical activity and diet quality with incident DM in the EPIC-Norfolk Study (*n* = 8,854, from 1993 to 2020). Estimates were adjusted for potential confounders as described in Model 2 in Methods, (A) comparing all trajectories (G1 as reference) and (B) with stratification by baseline exposure levels (stable behaviours as references). MDS^1^ = Mediterranean diet score at baseline assessment; PAEE^1^ = physical activity energy expenditure at baseline assessment, MDS^2^ = MDS at repeated assessment; PAEE^2^ = PAEE at repeated assessment; L = Low; H = High. High diet quality cut-off: MDS ≥ 8.5 points; high PA cut-off: PAEE ≥ 5 kJ/kg/day.

Joint associations of different trajectories of PA and diet quality with incident DM are shown in Fig. 1, based on Model 2. Panel A shows the results across all trajectories compared to the reference group with consistently low PAEE and low MDS (G1), revealing the strongest differences among participants who increased both behaviours during the exposure assessment period (G4, 50% lower risk), and participants who maintained both high PA and diet quality over time (G16, 40% lower risk). Other significant differences were observed in those who had sustained high diet quality despite decreasing their PA level over time (G15, 37% lower risk), and those who increased their diet quality over time while maintaining a high level of PA (G8, 31% lower risk). When stratified by baseline exposure level (Fig. 1, Panel B) and comparing to those who sustained this level at the repeated assessment, those increasing their activity and consistently eating a poor quality diet experienced 36% lower risk of DM (G2) but risk was 53% lower if also improving diet (G4). Starting high with either of the two exposures and then dropping to low, with or without improvement in the other behaviour at the repeat assessment was not associated with differences in risk (G5-G12). Starting high in both behaviours and decreasing diet quality was associated with higher risk of DM (G14, 60% higher risk), whereas the group who dropped both diet and activity (G13) had a 37% higher risk although not significant.

### Joint associations of trajectories of PA and diet quality with incidence of CVD

**Fig. 2:**
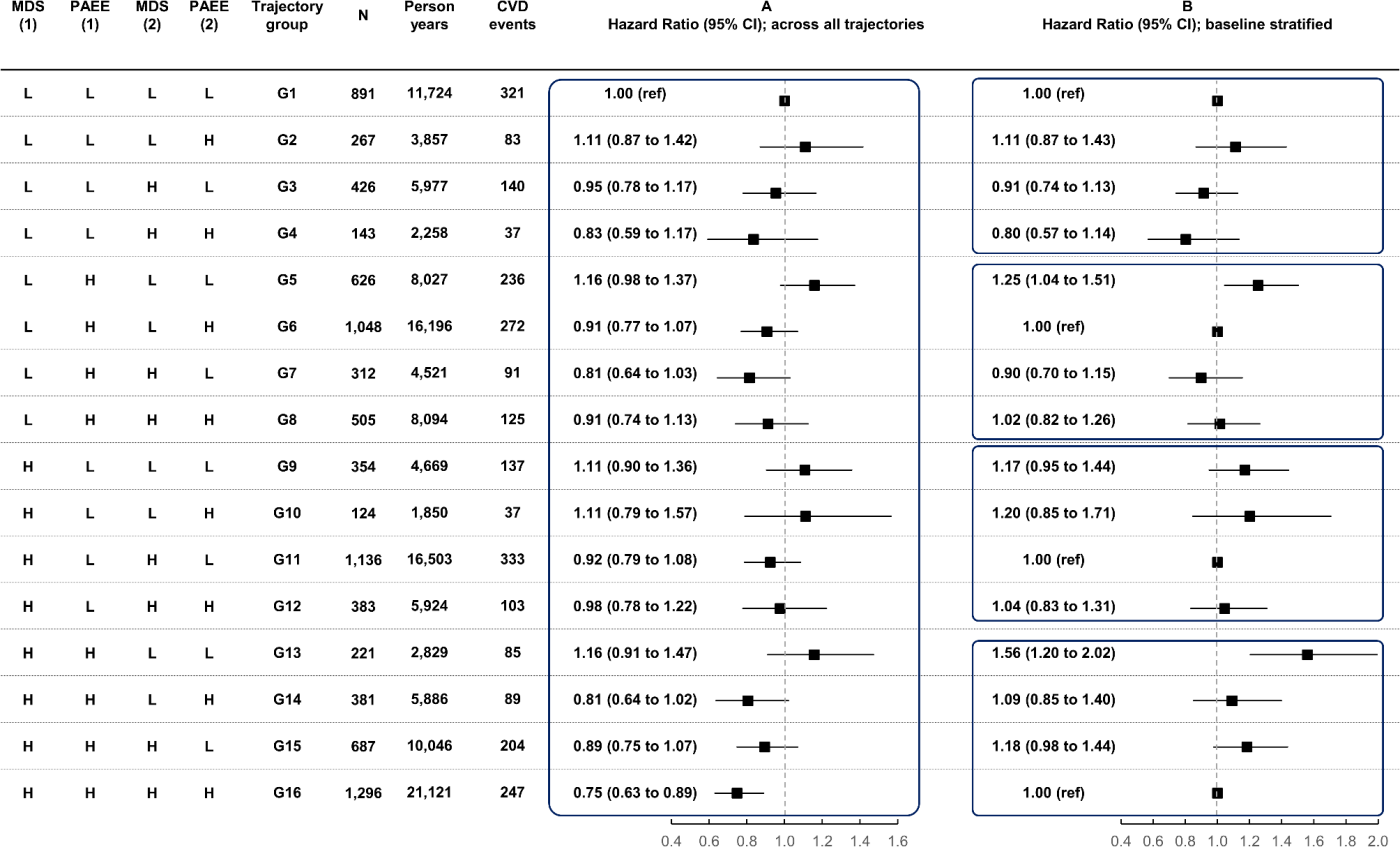
Joint associations of different trajectories of physical activity and diet quality with incident CVD in the EPIC-Norfolk Study (*n* = 8,800, from 1993 to 2022). Estimates were adjusted for potential confounders as described in Model 2 in Methods, (A) comparing all trajectories (G1 as reference) and (B) with stratification by baseline exposure levels (stable behaviours as references). MDS^1^ = Mediterranean diet score at baseline assessment; PAEE^1^ = physical activity energy expenditure at baseline assessment, MDS^2^ = MDS at repeated assessment; PAEE^2^ = PAEE at repeated assessment; L = Low; H = High. High diet quality cut-off: MDS ≥ 8.5 points; high PA cut-off: PAEE ≥ 5 kJ/kg/day.

Joint associations of different trajectories of PA and diet quality with incident CVD is shown in Fig. 2, based on Model 2. When all trajectory groups were compared to the reference group with sustained low PAEE and low MDS (G1), only those who maintained both high PA and diet quality over time had significantly lower risk of CVD (G16, 25% lower risk) (Fig. 2, Panel A). In stratified analyses with trajectory groups with stable behaviours considered as referents, only decreasing PA level (G5, 25% higher risk) and decreasing both PA and diet quality over time was significantly associated with higher risk of incident CVD (G13, 56% higher risk). The results slightly attenuated in Model 3 but still followed the same overall pattern (Figure S4 and Figure S5).

In ancillary analyses for trajectories based on three levels of exposures at baseline and at repeated assessments, separately for physical activity and diet (but mutually adjusted for the other), the lowest risk for incident DM was consistently for the highest exposure levels at baseline and repeated assessment (Figure S6, Panel A and Panel B). The greatest difference in incident CVD risk was observed in those moving from low exposure levels to medium exposure levels, with little or no additional risk difference between medium and high levels (Figure S6, Panel A and Panel B). For the joint activity-diet analysis of change, when considering the three categories of behaviour maintainers, decreasers and increasers, improvements in either or both exposures were associated with lower DM risk and CVD risk (Figure S64, Panel C).

### Population impact

**Fig. 3:**
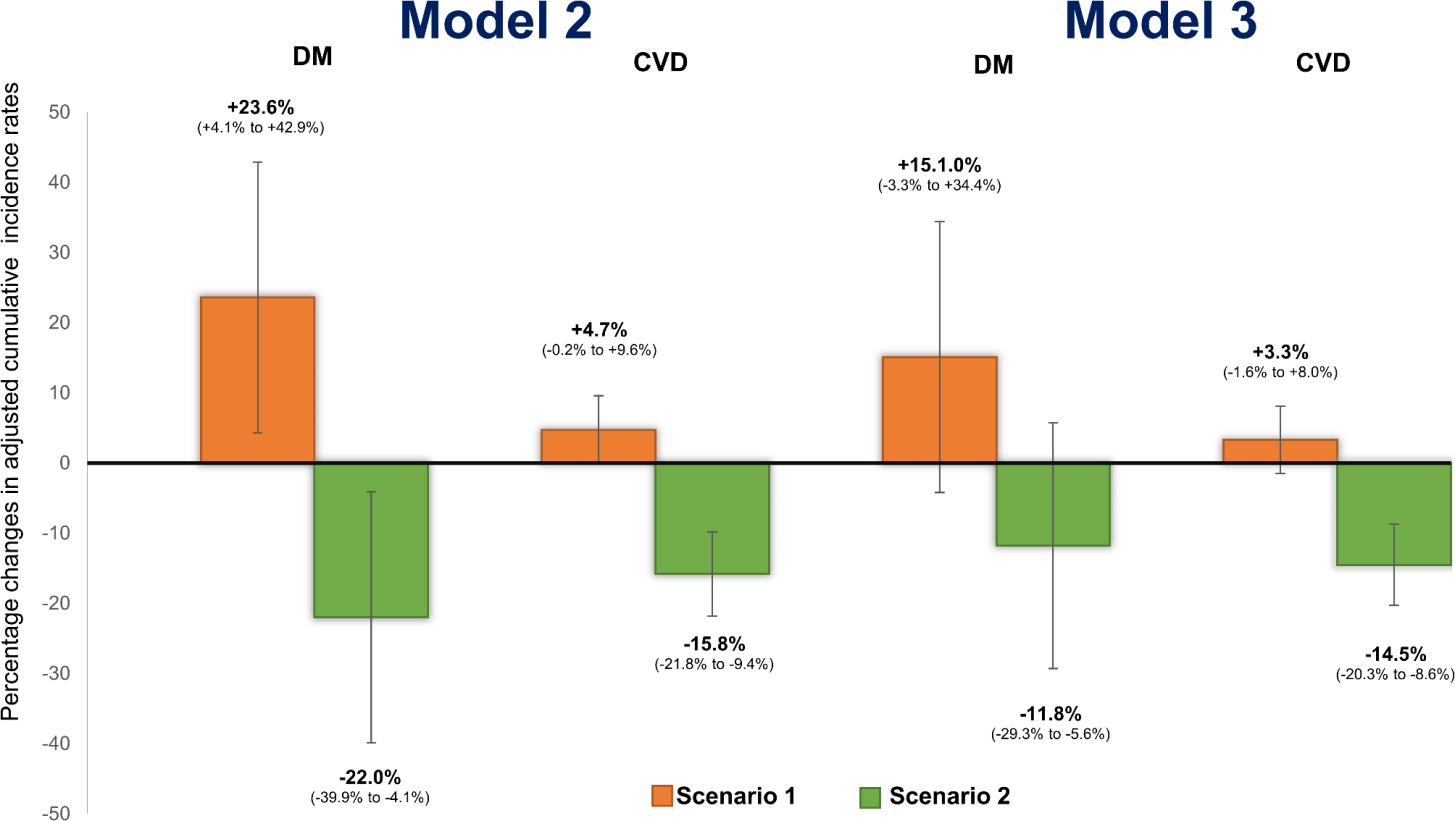
Population impact by estimating changes in adjusted cumulative incidence of cardiometabolic diseases (DM and CVD) in the population during two decades of follow-up, under two counterfactual scenarios. Counterfactual scenario 1; applying the adjusted cumulative incidence of trajectory group 1 (G1, low PA and low diet quality at both assessments) to the whole population. Counterfactual scenario 2; applying the adjusted cumulative incidence of trajectory group 16 (G16, high PA and high diet quality at both assessments) to the whole population. Model 3 adds adjustment for adiposity to Model 2. Error bars indicate 95% confidence intervals (95% CI) for percentage of differences in cumulative incidence under counterfactual scenarios compared with the observed scenarios.

Differences between observed and estimated cumulative incidence rates of DM and CVD were used to estimate population impact of adhering to sustained low PA and low diet quality (counterfactual scenario 1) or adhering to sustained high PA and high diet quality (counterfactual scenario 2) (Fig. 3 and Figure S7). Adjusted for potential confounders (Model 2), compared to the results under observed PA and diet exposures, cumulative incidence rates of DM and CVD would have been 23.6 (95% CI: 4.1 to 42.9)% and 4.7 (95% CI: -0.2 to 9.6)% higher, respectively if all the participants recorded low PA and low diet quality at both measurements. G16 made the largest contributions to the number of incident cases prevented for both diseases, followed by G15 for DM and G11 for CVD (Table S8 and Table S9). In contrast, had the whole population recorded high PA and high diet quality at both measurements, cumulative incidence rates of DM and CVD would have been 22.0 (95% CI: 4.1 to 39.9)% lower and 15.8 (95% CI: 9.4 to 21.8)% lower, respectively, with the largest contributions from G1 and G5.

## Discussion

### Principal findings

In this large prospective study with repeated exposure assessments, we examined the interplay between physical activity and diet quality in relation to DM and CVD risk. Our findings suggest that higher levels of these health behaviours at mid-adulthood and increases in them over time may lower the risk of DM and CVD. The greatest risk difference was observed when favourable levels of both exposures were combined, initiated earlier in life, and maintained over time. However, adopting an active lifestyle, a good quality diet, or both, even later in life, may improve cardiometabolic health. Interaction analyses suggest that achieving both leads to synergistic benefits.

Observed associations between within-person change in exposure and health outcomes are less likely to be confounded by factors that remain constant within individuals, such as genetic predispositions to diseases, since these factors are intrinsically controlled for. Furthermore, our findings of changes in physical activity and dietary behaviours were independent of their baseline levels. We observed evidence of reciprocity of within-person changes in physical activity and diet quality, in that participants starting from unfavourable levels but improving to favourable levels had 53% lower risk of DM and 20% lower risk of CVD, and conversely those who started off highly active and eating a good quality diet but then dropping to low levels for both had 37% and 56% higher risk of DM and CVD, respectively. Collectively, these observations strengthen the likelihood that physical activity and diet quality are causally related to cardiometabolic disease.

### Comparison with other studies

Previous observational studies have shown that an increase in PA level over time, compared to maintaining a stable state of PA, is linked to a decreased risk of diabetes and CVD. The direction of the association we observed aligns with findings from earlier studies^12,13,19,20,40–42^. However, in previous studies, the comparison involved a category transitioning from a low PA status to a high PA status, compared with a consistently low reference category to illustrate the impact of changes in PA over time. This lack of quantification of exposure change over time makes direct comparisons with our findings challenging. In our primary models, we employed continuous analysis to demonstrate the association of PA increase over time (per standard deviation unit, equivalent to ~ 5 kJ/kg/day during the assessment period) while adjusting for the baseline value of PA, resulting in 11% and 6% lower risk for DM and CVD, respectively This approach is considered more comprehensive as the categorisation of continuous variables results in the loss of information. Moreover, the ‘constant low PA category’ does not accurately represent all states of stable PA (e.g., stable high PA). Also, previous studies failed to account for diet quality and time-varying confounders in their models^12,13,19,20^, did not present results adjusted for adiposity markers^13,20,40^, had a shorter duration of follow-up after exposure assessment^13,19,20,41,42^, or were conducted in populations already at a high risk for development of the disease^40^, collectively, making their results more prone to potential overestimation of the effect size.

Associations of change in diet quality over time with CMD has been investigated in three U.S. prospective cohorts, showing 9% and 11% lower risk for incident diabetes per 10% increase^26,43^, and 4% lower risk for incident CVD per 20-percentile increase^21^. In contrast to our approach, these studies employed a distinct methodology for time analysis, dividing it into 4-year assessment periods and subsequent follow-up periods. They also adopted different diet quality indices, including plant-based diet indices (PBD), alternate healthy eating index (AHEI), and alternative MDS, as their exposure variables. Despite these variations, their findings consistently align with the results in our study, where each SD increase in ΔMDS was equivalent to 8.8% increase in diet quality over a 4-year period and associated with an 8% lower DM incidence and a 7% lower CVD incidence. In our study, we adopted a more comprehensive approach by considering concurrent changes in both PA and diet quality, examining the interaction between these factors and exploring shifts in health behaviours in opposite directions, concerning both DM and CVD as the outcomes.

The role of adiposity in the association between PA and dietary behaviours and CMD is complex. While adiposity can be considered a confounder of the relationship by altering engagement in activities and dietary habits and affecting the validity of self-reported behaviours, it may also be considered a mediator, since obesity is a known mechanism through which PA and diet influence the risk of DM and CVD^44–49^. Hence, not accounting for adiposity could lead to an overestimation of the association, while adjusting for adiposity may yield conservative estimates of the association independent of adiposity. For this reason, we presented results both without and with adjustment for BMI and WC. In line with existing literature^46,47,50^, the adjustment attenuated the associations, which was more pronounced when examining DM as the outcome than CVD. Nevertheless, the main findings and the public health implications drawn from these associations remained consistent.

### Strengths and limitations

With a prospective study design and incorporating repeated assessment of both PA and diet and multi-system health record linkage, we took a comprehensive approach to interrogating associations between these health behaviours and CMD. Specifically, we calibrated the self-reported activity measure to objective estimates of PAEE using our validation study, and our diet quality metric included 15 items of which 14 were assigned a continuous value, which is likely to have improved the validity of the diet-quality measurement. We took into account the independent and joint associations of health behaviours, and their trajectories as our exposures, considering their baseline levels as well as how they changed over time and interacted with each other. Our repeated measures analysis enabled separating between-person and within-person variations of exposures, enhancing the precision of the statistical tests. We adjusted our analyses for several static and time-varying confounding factors using a stepwise covariate model building approach, considering the role of adiposity markers as potential confounders or mediators in the associations. To comprehensively investigate the associations of the main exposures with CMD, we included several major disease endpoints including DM, total and fatal CVD, total and fatal IHD, and total and fatal stroke, ascertained via both internal data sources and data linkage of all participants to national registries during approximately two decades of follow-up after the exposure assessment period.

The study also has several limitations due to aspects of study design, though we conducted a number of sensitivity analyses to assess the robustness of our findings. The observational nature of our study, whilst stronger than single-assessment cohort studies, still limits firm conclusions on causality. The exclusion of a large proportion of baseline participants (63.8%) due to the unavailability of repeated assessments may have introduced selection bias. The included sample had higher baseline physical activity and diet quality levels and lower cardiometabolic risk factors compared to the excluded sample (data not shown). However, this does not influence internal validity of our findings, but rather impacts their generalisability. Since we analysed a healthier sample, the associations we observed might have been more pronounced in the entire study sample. Moreover, the association of baseline exposures with outcomes in the entire EPIC-Norfolk cohort were similar to those observed in the sub-cohort used for the present analyses, suggesting the natural depletion of the sample over time did not materially introduce selection bias in relative associations. Although validated, subjective self-reported assessment of PA and diet are susceptible to recall bias and social desirability bias which might bias associations in either direction. The possibility of imprecisely measured or unmeasured factors, leaves open the possibility of insufficiently adjusted associations because of residual confounding. Ideally, a long-term large randomised controlled trial is necessary to overcome such limitations. However, that is impractical with regard to altering health behaviours including the physical nature of people`s jobs and accumulating disease events over a sustained period of time as well as the logistics and cost of conducting such a trial. We acknowledge that our data did not allow distinguishing subtypes of DM, particularly Type 1 and 2. However, considering the age group of our participants and the very low incidence rate for Type 1 DM in this cohort, this limitation would not impact our study findings. A potential limitation of our trajectory analysis is that some of the 16 trajectory groups have small sample sizes, which could limit statistical power for detecting associations with outcomes. However, our primary analysis examines physical activity and diet quality as continuous variables which is free from this issue; and we also conducted an ancillary analysis using 9 joint trajectory groups to mitigate this potential problem. While our exploratory analyses (Figure S2) support that the dose-response relationships can be reasonably approximated using linear functions, the association between PAEE and CVD appear slightly more curvilinear, with steeper associations at the lower end of PAEE. However, the linear modelling approach of four mutually adjusted exposures of activity and diet quality yields easier-to-interpret estimates of association. Another potential limitation is the risk of detecting false associations due to multiple testing; however, we followed our pre-specified analysis plan and present magnitude of association alongside their confidence limits rather than isolated significant results. Finally, the EPIC-Norfolk study largely includes middle-aged and older, mostly white European participants residing in the East of England, and our findings may not generalise to other populations, particularly those with a different socioeconomic or occupational position, food supply chain, and transport systems.

### Public health implication

We estimated that ~ 22% of DM events and ~ 16% of CVD events could have been prevented with sustained adherence to higher levels of PA and diet quality in this population. This can be accomplished, for example by 30 min per day of brisk walking or other exercise, alongside reducing red meat consumption to less than twice a week, increasing fish consumption to at least once a week, and vegetable consumption to at least three servings per day. We identified large subgroups with suboptimal levels of one or both of these health behaviours at different times during adulthood. Considering the potential synergistic effects between PA, diet quality, and their changes over time in relation to the CMD development, we advocate for promoting the adoption of being physically active combined with a healthy diet throughout adulthood to improve cardiometabolic health at the population level.

The interactions we observed suggest that improving both physical activity and diet quality together may lead to a greater reduction in CMD risk than expected from improving either behaviour alone. This highlights the importance of integrated lifestyle interventions that promote simultaneous changes in physical activity and diet quality, rather than focusing on single-behaviour modifications, to maximise cardiovascular disease prevention at the population level.

## Conclusions

In summary, we have demonstrated that improving both PA and diet quality is relevant for cardiometabolic health. Considering the association of both the starting point and trajectory of these health behaviours with CMD, initiating a healthy lifestyle sooner seems to be more beneficial. Nevertheless, it is important to recognise that embarking the transition to a healthy lifestyle remains favourable for health even in mid or late adulthood.

## Electronic supplementary material

Below is the link to the electronic supplementary material.

Supplementary Material 1

## Data Availability

The data that support the findings of this study are not publicly available under the EPIC-Norfolk study data ethics and policy. However, data are available upon reasonable request to and approval by the EPIC-Norfolk Management Committee (epic-norfolk@mrc-epid.cam.ac.uk).
