# Supplementary material for: Combined associations of physical activity, diet quality and their trajectories with incidence of diabetes and cardiovascular diseases in the EPIC-Norfolk Study
Source: Sci Rep. 2025 Apr 16;15:11261. doi: 10.1038/s41598-025-93679-x (PMC12003676; doi:10.1038/s41598-025-93679-x)
Supplement: Supplementary file 1 — Supplementary Material 1 [file 41598_2025_93679_MOESM1_ESM.pdf]

Supplementary Material for Aryannezhad et al

Combined associations of physical activity, diet quality and their trajectories with incidence of diabetes and cardiovascular diseases: the EPIC-Norfolk Study

LIST OF CONTENT

Figure S1 - Flow diagram illustrating selection of the analytical sample	Page 2
Table S1 - Hazard ratios with 95% confidence intervals for the associations of mutually adjusted exposures with subtypes of cardiovascular diseases	Page 3
Table S2 - Associations of exposures with incident cardiometabolic diseases, mutually adjusted for potential confounders and mediators (Model 4)	Page 4
Table S3 - Associations of mutually adjusted exposures with incidence of DM and CVD (complete case analysis)	Page 5
Table S4 - Hazard ratios with 95% confidence intervals for the associations of mutually adjusted exposures with incident DM and CVD, considering health check 3 as the end of the assessment period	Page 6
Table S5 - Hazard ratios with 95% confidence intervals for the associations of mutually adjusted exposures with incident DM and CVD, excluding events that occurred within two years of the last measurement	Page 7
Table S6 - Associations between baseline exposures and the outcomes DM and CVD in the entire EPIC cohort, compared to the sub-sample with repeat assessments.	Page 8
Table S7 - Tests of interaction between different combinations of the exposures for incidence of DM and CVD	Page 9
Figure S2 - Dose-response relationship between PA and diet exposures and incidence of DM (Panel A) and CVD (Panel B) fitted by using a Cox Proportional-Hazards with fractional polynomial	Page 10
Figure S3 – Associations of mutually adjusted baseline and within-person changes in PAEE and MDS with incident DM and incident CVD in different strata of baseline age, sex, BMI, and smoking	Page 11
Figure S4 -Joint associations of different trajectories of physical activity and diet quality with incident; Analysis based on Model 3	Page 12
Figure S5 -Joint associations of different trajectories of physical activity and diet quality with incident DM; Analysis based on Model 3	Page 13
Figure S6 - Associations of different trajectories of PA and diet with incidence of DM and CVD, based on three-by-three levels of exposures	Page 14
Figure S7 - Population impact by estimating changes in cumulative adjusted incident rates of any CMD (DM or CVD) and subtypes of CVD in the population during two decades of follow-up	Page 15
Table S8- Population impact by estimating the differences in total number of DM cases that could have been potentially observed under two different counterfactual scenarios.	Page 16
Table S9- Population impact by estimating the differences in total number of CVD cases that could have been potentially observed under two different counterfactual scenarios	Page 17

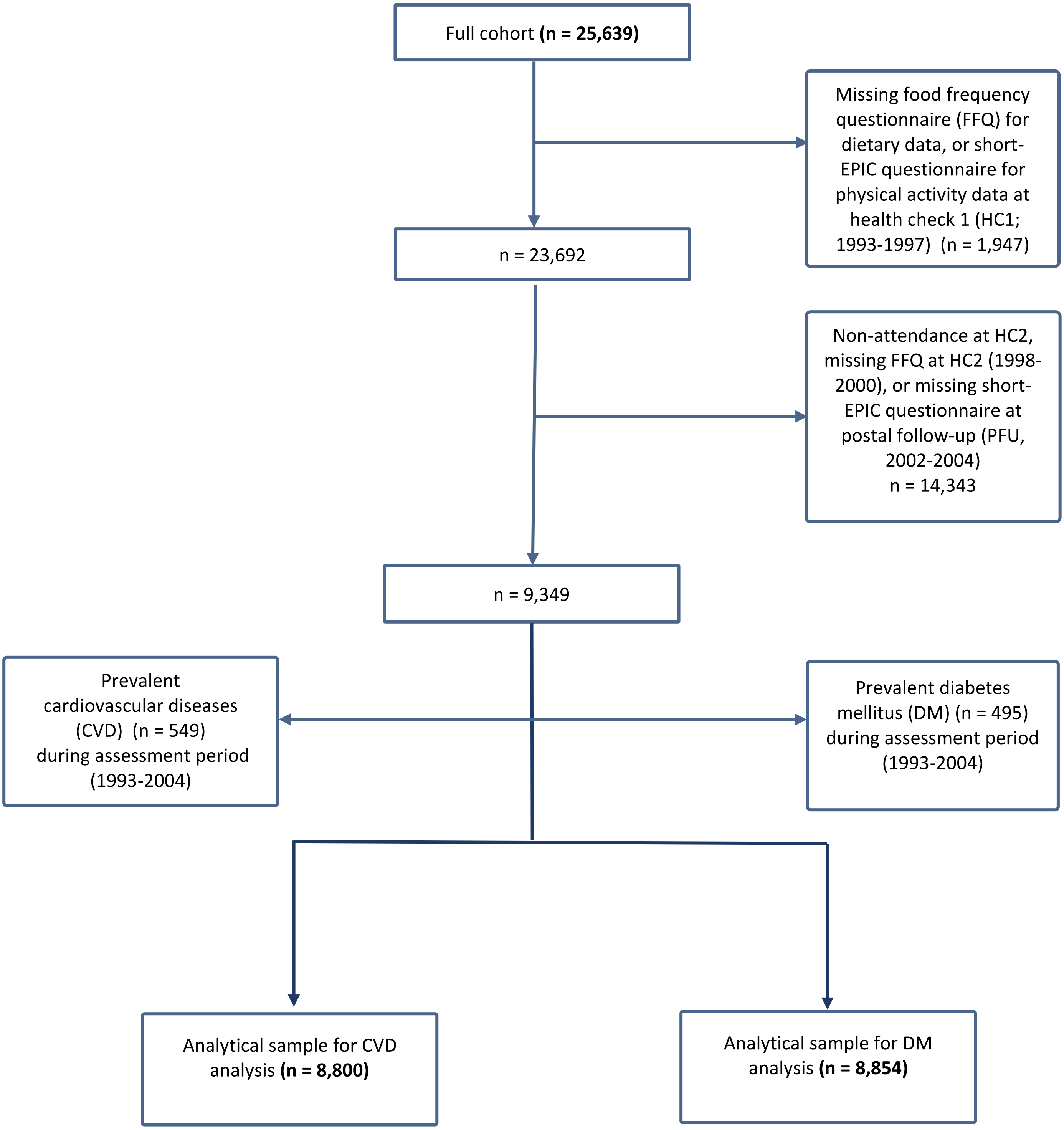

Figure S1 - Flow diagram illustrating selection of the analytical sample in the EPIC-Norfolk Study.

Table S1 - Hazard ratios with 95% confidence intervals for the associations of mutually adjusted exposures with subtypes of cardiovascular diseases in the EPIC-Norfolk Study†

**Outcome**	**Exposures‡**	**Model 1 (minimally adjusted)**	**Model 2 (further adjustment for potential confounders)**	**Model 3 (further adjustment for adiposity)**
**Incident fatal CVD**<br>1,513 cases<br>129,484 person-years	Baseline PAEE	0.89 (0.83 to 0.94)	0.91 (0.85 to 0.97)	0.92 (0.86 to 0.98)
	ΔPAEE	0.86 (0.81 to 0.92)	0.88 (0.82 to 0.94)	0.89 (0.83 to 0.95)
	Baseline MDS	0.89 (0.84 to 0.94)	0.90 (0.85 to 0.96)	0.91 (0.85 to 0.97)
	ΔMDS	0.88 (0.83 to 0.93)	0.89 (0.84 to 0.94)	0.89 (0.84 to 0.95)
**Incident IHD**<br>1,622 cases<br>132,615 person-years	Baseline PAEE	0.91 (0.85 to 0.96)	0.93 (0.87 to 0.99)	0.95 (0.89 to 1.01)
	ΔPAEE	0.93 (0.88 to 0.99)	0.95 (0.90 to 1.01)	0.96 (0.90 to 1.02)
	Baseline MDS	0.96 (0.91 to 1.02)	0.97 (0.92 to 1.03)	0.98 (0.92 to 1.04)
	ΔMDS	0.93 (0.88 to 0.99)	0.94 (0.89 to 1.00)	0.95 (0.90 to 1.01)
**Incident fatal IHD**<br>910 cases<br>132,615 person-years	Baseline PAEE	0.86 (0.79 to 0.94)	0.89 (0.82 to 0.97)	0.91 (0.83 to 0.99)
	ΔPAEE	0.86 (0.79 to 0.94)	0.89 (0.81 to 0.97)	0.89 (0.82 to 0.97)
	Baseline MDS	0.91 (0.84 to 0.98)	0.93 (0.85 to 1.01)	0.93 (0.86 to 1.01)
	ΔMDS	0.88 (0.82 to 0.95)	0.89 (0.83 to 0.96)	0.90 (0.84 to 0.97)
**Incident stroke**<br>1,295 cases<br>138,504 person-years	Baseline PAEE	0.92 (0.86 to 0.98)	0.93 (0.87 to 1.00)	0.94 (0.87 to 1.01)
	ΔPAEE	0.94 (0.87 to 1.00)	0.95 (0.89 to 1.02)	0.95 (0.89 to 1.02)
	Baseline MDS	0.90 (0.85 to 0.96)	0.89 (0.83 to 0.96)	0.89 (0.83 to 0.95)
	ΔMDS	0.89 (0.84 to 0.95)	0.88 (0.83 to 0.94)	0.88 (0.83 to 0.94)
**Incident fatal stroke**<br>851 cases<br>138,504 person-years	Baseline PAEE	0.92 (0.84 to 1.00)	0.94 (0.86 to 1.02)	0.94 (0.86 to 1.03)
	ΔPAEE	0.90 (0.82 to 0.98)	0.91 (0.84 to 1.00)	0.91 (0.84 to 1.00)
	Baseline MDS	0.86 (0.80 to 0.93)	0.86 (0.79 to 0.94)	0.86 (0.79 to 0.94)
	ΔMDS	0.86 (0.79 to 0.92)	0.85 (0.79 to 0.92)	0.85 (0.78 to 0.92)

†HRs per SD difference in each exposure are presented. Sample size n=8,800 for CVD outcomes (end of follow-up: March 2022). All coefficients are mutually adjusted for the four primary exposures (baseline PAEE, ΔPAEE, baseline MDS, ΔMDS). Covariates in the Models: Model 1: sex, age. Model 2: variables in Model 1 + education, social class, marital status, FH of DM, FH of MI; and time updated variables for smoking, HRT, total energy intake, lipid-lowering drugs, antihypertensive drugs, anti-diabetes drugs, prevalent DM. Model 3: variables in Model 2 + time updated variables for BMI, WC. ‡ SD increment in baseline PAEE equals to 4.64 kJ/kg/day, in ΔPAEE equals to 0.65 kJ/kg/day per year, in baseline MDS equals to 1.30 points, and in ΔMDS equals to 0.33 points per year. Abbreviations: CI, Confidence interval; CVD, cardiovascular diseases; EPIC, European Prospective Investigation of Cancer and nutrition; HR, hazard ratio; MDS, Mediterranean diet score; PAEE, physical activity energy expenditure; SD, standard deviation.

Table S2 - Associations of exposures with incident cardiometabolic diseases in the EPIC-Norfolk Study, mutually adjusted for potential confounders and mediators (Model 4)†

Outcome	Exposures‡	HR (95% CI)
Incident DM<br>968 cases<br>123,036 person-years	Baseline PAEE	0.97 (0.89 to 1.05)
	ΔPAEE	0.92 (0.85 to 0.99)
	Baseline MDS	0.92 (0.85 to 1.00)
	ΔMDS	0.95 (0.89 to 1.03)
Incident CVD<br>2,540 cases<br>129,484 person-years	Baseline PAEE	0.94 (0.90 to 0.99)
	ΔPAEE	0.95 (0.90 to 1.00)
	Baseline MDS	0.95 (0.90 to 1.00)
	ΔMDS	0.93 (0.89 to 0.97)
Incident fatal CVD<br>1,513 cases<br>129,484 person-years	Baseline PAEE	0.92 (0.86 to 0.99)
	ΔPAEE	0.89 (0.83 to 0.95)
	Baseline MDS	0.92 (0.86 to 0.98)
	ΔMDS	0.89 (0.84 to 0.95)
Incident IHD<br>1,622 cases<br>132,615 person-years	Baseline PAEE	0.95 (0.90 to 1.01)
	ΔPAEE	0.97 (0.91 to 1.03)
	Baseline MDS	0.98 (0.93 to 1.04)
	ΔMDS	0.95 (0.90 to 1.01)
Incident fatal IHD<br>910 cases<br>132,615 person-years	Baseline PAEE	0.91 (0.84 to 0.99)
	ΔPAEE	0.90 (0.82 to 0.98)
	Baseline MDS	0.94 (0.86 to 1.02)
	ΔMDS	0.90 (0.83 to 0.97)
Incident stroke<br>1,295 cases<br>138,504 person-years	Baseline PAEE	0.94 (0.87 to 1.01)
	ΔPAEE	0.95 (0.88 to 1.02)
	Baseline MDS	0.90 (0.84 to 0.96)
	ΔMDS	0.88 (0.83 to 0.94)
Incident fatal stroke<br>851 cases<br>138,504 person-years	Baseline PAEE	0.94 (0.86 to 1.03)
	ΔPAEE	0.92 (0.84 to 1.00)
	Baseline MDS	0.87 (0.80 to 0.94)
	ΔMDS	0.85 (0.79 to 0.92)

†HRs per SD difference in each exposure are presented. Sample size n=8,854 for DM outcome (end of follow-up: March 2020) and n=8,800 for CVD outcomes (end of follow-up: March 2022). All coefficients are mutually adjusted for the four primary exposures (baseline PAEE, ΔPAEE, baseline MDS, ΔMDS). Covariates in the Model 4: sex, age, education, social class, marital status, FH of DM, FH of MI, time updated variables for smoking, HRT, total energy intake, lipid-lowering drugs, antihypertensive drugs, anti-diabetes drugs (only when CVD is the outcome), prevalent diseases (prevalent CVD when DM is the outcome, prevalent DM when CVD is the outcome, BMI, WC, TG, LDL, HDL, SBP, and DBP.

‡ SD increment in baseline PAEE equals to 4.64 kJ/kg/day, in ΔPAEE equals to 0.65 kJ/kg/day per year, in baseline MDS equals to 1.30 points, and in ΔMDS equals to 0.33 points per year.

Abbreviations: CI, Confidence interval; CVD, cardiovascular diseases; EPIC, European Prospective Investigation of Cancer and nutrition; HR, hazard ratio; MDS, Mediterranean diet score; PAEE, physical activity energy expenditure; SD, standard deviation.

Table S3 - Associations of mutually adjusted exposures with incidence of DM and CVD in the EPIC-Norfolk Study (complete case analysis)†

	Exposures††	Model 1 (minimally adjusted)	Model 2 (further adjustment for potential confounders)	Model 3 (further adjustment for adiposity)	Model 4 (further adjustment for potential mediators)
No. of participants		8,854	8,591	8,560	7,620
Time at risk		123,036	119,604	119141	106680
**Incident DM**		968	934	932	774
	Baseline PAEE	0.87 (0.80 to 0.94)	0.89 (0.82 to 0.97)	0.96 (0.89 to 1.04)	0.98 (0.90 to 1.07)
	ΔPAEE	0.85 (0.79 to 0.92)	0.88 (0.81 to 0.95)	0.91 (0.84 to 0.98)	0.90 (0.83 to 0.99)
	Baseline MDS	0.85 (0.79 to 0.91)	0.89 (0.82 to 0.96)	0.91 (0.85 to 0.99)	0.93 (0.85 to 1.01)
	ΔMDS	0.90 (0.84 to 0.96)	0.92 (0.86 to 1.00)	0.96 (0.89 to 1.04)	0.96 (0.88 to 1.04)
No. of participants		8,800	8,530	8,498	7,557
Time at risk		129,484	125590	125090	111393
**Incident CVD**		2,540	2,464	2,457	2,185
	Baseline PAEE	0.91 (0.87 to 0.95)	0.93 (0.89 to 0.98)	0.94 (0.90 to 0.99)	0.94 (0.89 to 0.99)
	ΔPAEE	0.92 (0.88 to 0.97)	0.94 (0.89 to 0.98)	0.94 (0.89 to 0.99)	0.94 (0.89 to 0.99)
	Baseline MDS	0.93 (0.89 to 0.98)	0.94 (0.90 to 0.99)	0.95 (0.90 to 1.00)	0.96 (0.91 to 1.01)
	ΔMDS	0.92 (0.88 to 0.96)	0.93 (0.89 to 0.97)	0.93 (0.89 to 0.98)	0.95 (0.90 to 1.00)

†HRs per SD difference in each exposure are presented for DM outcome end of follow-up: March 2020; for CVD outcomes end of follow-up: March 2022. All coefficients are mutually adjusted for the four primary exposures (baseline PAEE, ΔPAEE, baseline MDS, ΔMDS). Covariates in the Models: Model 1: sex, age. Model 2: variables in Model 1 + education, social class, marital status, FH of DM, FH of MI; and time updated variables for smoking, HRT, total energy intake, lipid-lowering drugs, antihypertensive drugs, anti-diabetes drugs (only when CVD is the outcome), prevalent diseases (prevalent CVD when DM is the outcome, prevalent DM when CVD is the outcome). Model 3: variables in Model 2 + time updated variables for BMI, WC. Model 4: variables in Model 3 + time updated variables for TG, LDL, HDL, SBP, and DBP.

‡ SD increment in baseline PAEE equals to 4.64 kJ/kg/day, in ΔPAEE equals to 0.65 kJ/kg/day per year, in baseline MDS equals to 1.30 points, and in ΔMDS equals to 0.33 points per year.

Abbreviations: CI, Confidence interval; CVD, cardiovascular diseases; EPIC, European Prospective Investigation of Cancer and nutrition; HR, hazard ratio; MDS, Mediterranean diet score; PAEE, physical activity energy expenditure; SD, standard deviation.

Table S4 - Hazard ratios with 95% confidence intervals for the associations of mutually adjusted exposures with incident DM and CVD in the EPIC-Norfolk Study, considering health check 3 as the end of the assessment period †

Outcome	Exposures‡	Model 1 (minimally adjusted)	Model 2 (further adjustment for potential confounders)	Model 3 (further adjustment for adiposity)
**Incident DM**<br>488 cases<br>54001 person-years	Baseline PAEE	0.93 (0.83 to 1.04)	0.95 (0.85 to 1.07)	1.01 (0.90 to 1.14)
	ΔPAEE	0.91 (0.81 to 1.01)	0.92 (0.82 to 1.03)	0.95 (0.85 to 1.06)
	Baseline MDS	0.91 (0.83 to 1.00)	0.96 (0.86 to 1.06)	0.97 (0.87 to 1.08)
	ΔMDS	0.95 (0.86 to 1.04)	0.97 (0.88 to 1.07)	0.98 (0.89 to 1.08)
**Incident CVD**<br>1,217 cases<br>59246 person-years	Baseline PAEE	0.93 (0.86 to 1.00)	0.94 (0.87 to 1.01)	0.95 (0.88 to 1.02)
	ΔPAEE	0.94 (0.87 to 1.01)	0.94 (0.87 to 1.01)	0.95 (0.88 to 1.02)
	Baseline MDS	0.96 (0.90 to 1.02)	0.96 (0.90 to 1.03)	0.97 (0.90 to 1.03)
	ΔMDS	0.98 (0.93 to 1.05)	0.99 (0.93 to 1.05)	0.99 (0.93 to 1.06)

†HRs per SD difference in each exposure are presented. Sample size n=5,493 for DM outcome (end of follow-up: March 2020) and n=5,332 for CVD outcomes (end of follow-up: March 2022). All coefficients are mutually adjusted for the four primary exposures (baseline PAEE, ΔPAEE, baseline MDS, ΔMDS). Covariates in the Models: Model 1: sex, age. Model 2: variables in Model 1 + education, social class, marital status, FH of DM, FH of MI; and time updated variables for smoking, HRT, total energy intake, lipid-lowering drugs, antihypertensive drugs, anti-diabetes drugs (only when CVD is the outcome), prevalent diseases (prevalent CVD when DM is the outcome, prevalent DM when CVD is the outcome). Model 3: variables in Model 2 + time updated variables for BMI, WC. ‡ SD increment in baseline PAEE equals to 4.64 kJ/kg/day, in ΔPAEE equals to 0.65 kJ/kg/day per year, in baseline MDS equals to 1.30 points, and in ΔMDS equals to 0.33 points per year.

Abbreviations: CI, Confidence interval; CVD, cardiovascular diseases; EPIC, European Prospective Investigation of Cancer and nutrition; HR, hazard ratio; MDS, Mediterranean diet score; PAEE, physical activity energy expenditure; SD, standard deviation.

Table S5 - Hazard ratios with 95% confidence intervals for the associations of mutually adjusted exposures with incident DM and CVD in the EPIC-Norfolk Study, excluding events that occurred within two years of the last measurement †

Outcome	Exposures‡	Model 1 (minimally adjusted)	Model 2 (further adjustment for potential confounders)	Model 3 (further adjustment for adiposity)
**Incident DM**<br>Cases 921<br>person-years 122,839	Baseline PAEE	0.87 (0.81 to 0.95)	0.89 (0.82 to 0.97)	0.95 (0.88 to 1.03)
	ΔPAEE	0.86 (0.80 to 0.93)	0.88 (0.81 to 0.95)	0.91 (0.84 to 0.98)
	Baseline MDS	0.86 (0.80 to 0.92)	0.90 (0.83 to 0.97)	0.92 (0.85 to 1.00)
	ΔMDS	0.89 (0.83 to 0.96)	0.92 (0.85 to 0.99)	0.95 (0.88 to 1.03)
**Incident CVD**<br>Cases 2,297<br>person-years 129,123	Baseline PAEE	0.91 (0.87 to 0.96)	0.93 (0.88 to 0.98)	0.94 (0.90 to 0.99)
	ΔPAEE	0.94 (0.89 to 0.99)	0.95 (0.90 to 1.00)	0.96 (0.91 to 1.01)
	Baseline MDS	0.94 (.892 to 0.98)	0.94 (0.90 to 0.99)	0.94 (0.90 to 0.99)
	ΔMDS	0.91 (0.88 to 0.95)	0.92 (0.87 to 0.96)	0.92 (0.88 to 0.97)

†HRs per SD difference in each exposure are presented. Sample size n= 8,684 for DM outcome (end of follow-up: March 2020) and n=8,472 for CVD outcomes (end of follow-up: March 2022). All coefficients are mutually adjusted for the four primary exposures (baseline PAEE, ΔPAEE, baseline MDS, ΔMDS). Covariates in the Models: Model 1: sex, age. Model 2: variables in Model 1 + education, social class, marital status, FH of DM, FH of MI; and time updated variables for smoking, HRT, total energy intake, lipid-lowering drugs, antihypertensive drugs, anti-diabetes drugs (only when CVD is the outcome), prevalent diseases (prevalent CVD when DM is the outcome, prevalent DM when CVD is the outcome). Model 3: variables in Model 2 + time updated variables for BMI, WC. ‡ SD increment in baseline PAEE equals to 4.64 kJ/kg/day, in ΔPAEE equals to 0.65 kJ/kg/day per year, in baseline MDS equals to 1.30 points, and in ΔMDS equals to 0.33 points per year.

Abbreviations: CI, Confidence interval; CVD, cardiovascular diseases; EPIC, European Prospective Investigation of Cancer and nutrition; HR, hazard ratio; MDS, Mediterranean diet score; PAEE, physical activity energy expenditure; SD, standard deviation.

Table S6 - Associations between baseline exposures and the outcomes DM and CVD in the entire EPIC cohort, compared to the sub-sample with repeat assessments.

**Outcome**	**Hazard ratio (95% CI)**			
	Entire EPIC-Norfolk cohort		Sub-sample with repeat assessments	
**Incident DM**	n = 22,752<br>3,305 cases<br>436,234 person-years	Baseline PAEE:<br>0.92 (0.89 to 0.96)	n = 9,101<br>1,258 cases<br>189,690.432 person-years	Baseline PAEE:<br>0.91 (0.86 to 0.97)
		Baseline MDS:<br>0.91 (0.88 to 0.94)		Baseline MDS:<br>0.92 (0.86 to 0.97)
**Incident CVD**	n = 23,298<br>8,023 cases<br>458,640 person-years	Baseline PAEE: :<br>0.93 (0.90 to 0.95)	n = 9,245<br>3,031 cases<br>197,432 person-years	Baseline PAEE:<br>0.93 (0.90 to 0.97)
		Baseline MDS:<br>0.96 (0.94 to 0.98)		Baseline MDS:<br>0.99 (0.95 to 1.03)

Hazard Ratios per SD difference in each exposure
Multivariable Cox models are mutually adjusted for the two main exposures, as well as baseline sex, age, marital status, education level, social class, smoking, HRT, total energy intake

Table S7 - Tests of interaction between different combinations of the exposures for incidence of DM and CVD in the EPIC-Norfolk Study†

	Interaction terms in the model	Multiplicative interaction	Additive interaction
		**HR (95% CI)**	**RERI (95% CI)**
DM	a. Baseline PAEE × ΔPAEE	0.97 (0.92 to 1.03)	0.10 (-0.18 to 0.38)
	b. Baseline MDS × ΔMDS	1.00 (0.94 to 1.06)	0.05 (-0.13 to 0.23)
	c. Baseline PAEE × Baseline MDS	0.98 (0.92 to 1.05)	0.18 (-0.02 to 0.37)
	d. ΔPAEE × ΔMDS	1.00 (0.93 to 1.06)	0.13 (-0.06 to 0.32)
		**Likelihood-ratio test (p-value)††**	
	a + b	0.653	
	c + d	0.885	
	a + b + c + d	0.892	
		**Multiplicative interaction**	**Additive interaction**
		**HR (95% CI)**	**RERI (95% CI)**
CVD	a. Baseline PAEE × ΔPAEE	0.96 (0.93 to 0.99)*	0.05 (-0.11 to 0.21)
	b. Baseline MDS × ΔMDS	0.968 (0.934 to 1.00)	0.09 (-0.021 to 0.21)
	c. Baseline PAEE × Baseline MDS	0.967 (0.929 to 1.01)	0.11 (0.00 to 0.23)*
	d. ΔPAEE × ΔMDS	0.993 (0.953 to 1.03)	0.16 (0.04 to 0.28)*
		**Likelihood-ratio test (p-value)††**	
	a + b	0.018	
	c + d	0.268	
	a + b + c + d	0.032	

HR, Hazard ratio; CI, Confidence interval RERI, the relative excess risk due to interaction
†Adjusted for covariates in Model 2: sex, age, education, social class, marital status, FH of DM, FH of MI; and time updated variables for smoking, HRT, total energy intake, lipid-lowering drugs, antihypertensive drugs, anti-diabetes drugs (only when CVD is the outcome), prevalent diseases (prevalent CVD when DM is the outcome, prevalent DM when CVD is the outcome)
††Likelihood ratio test was used to assess the interaction when more than one interaction term was included in the model

***Statistically significant interaction terms**		**Stratifier**	**Exposure: HR (95% CI)**
a. Baseline PAEE × ΔPAEE for CVD	Baseline PAEE	< 5 kJ/kg/day	ΔPAEE, 1 SD increase: 1.02 (0.94 to 1.10)
		≥ 5 kJ/kg/day	ΔPAEE, 1 SD increase: 0.92 (0.87 to 0.97)
c. Baseline PAEE × Baseline MDS for CVD	Baseline PAEE	< 5 kJ/kg/day	Baseline MDS, 1 SD increase: 0.98 (0.91 to 1.05)
		≥ 5 kJ/kg/day	Baseline MDS, 1 SD increase: 0.91 (0.85 to 0.97)
d. ΔPAEE × ΔMDS for CVD	ΔPAEE	< 0 kJ/kg/day per year	ΔMDS, 1 SD increase: 0.94 (0.88 to 1.01)
		≥ 0 kJ/kg/day per year	ΔMDS, 1 SD increase: 0.92 (0.87 to 0.97)

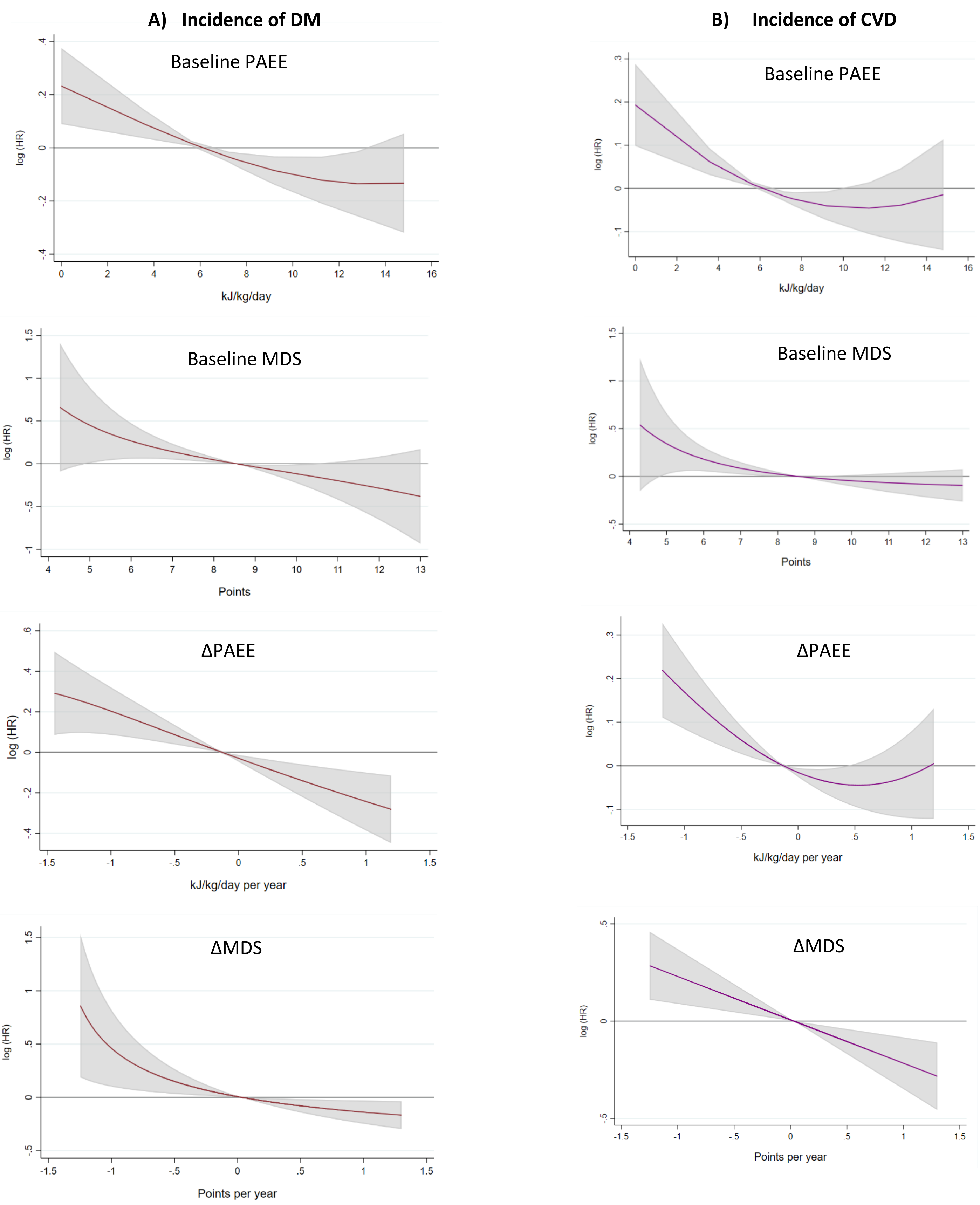

Figure S2 – Dose-response relationship between PA and diet exposures and incidence of DM (Panel A) and CVD (Panel B) fitted by using a Cox Proportional-Hazards with fractional polynomial in the EPIC-Norfolk Study.

Model 2 was used for this analysis (see methods).
The 95% CI is depicted in the shaded regions.
MDS=Mediterranean diet score; PAEE=physical activity energy expenditure; ΔMDS=over time changes in MDS; ΔPAEE=over time changes in PAEE.

A) Diabetes mellitus

Subgroups		N	DM events	Baseline PAEE HR (95%CI)	P†	ΔPAEE HR (95%CI)	P†	Baseline MDS HR (95%CI)	P†	ΔMDS HR (95%CI)	P†
All participants		8,854	968	0.89 (0.82 to 0.96)		0.87 (0.81 to 0.94)		0.89 (0.82 to 0.96)		0.92 (0.86 to 0.99)	
Age	<65 years	6,637	721	0.86 (0.79 to 0.94)	0.83	0.87 (0.80 to 0.95)	0.42	0.90 (0.82 to 0.98)	0.99	0.94 (0.86 to 1.02)	0.39
	≥65 years	2,217	247	0.87 (0.73 to 1.04)		0.79 (0.66 to 0.95)		0.86 (0.74 to 1.01)		0.86 (0.74 to 1.00)	
Sex	Male	3,598	443	0.88 (0.79 to 0.99)	0.79	0.90 (0.81 to 1.01)	0.22	0.87 (0.78 to 0.98)	0.44	0.90 (0.81 to 1.00)	0.54
	Female	5,256	525	0.90 (0.80 to 1.01)		0.85 (0.76 to 0.95)		0.91 (0.82 to 1.01)		0.94 (0.85 to 1.04)	
BMI	<25 kg/m²	3,951	223	0.93 (0.79 to 1.10)	0.16	0.88 (0.74 to 1.04)	0.18	0.87 (0.74 to 1.02)	0.98	0.80 (0.69 to 0.94)	0.05
	≥25 kg/m²	4,903	745	0.90 (0.82 to 0.99)		0.88 (0.81 to 0.96)		0.91 (0.83 to 1.00)		0.97 (0.89 to 1.05)	
Smoking	Current smokers	700	93	0.90 (0.69 to 1.17)	0.56	0.97 (0.75 to 1.25)	0.18	0.85 (0.66 to 1.08)	0.47	1.00 (0.80 to 1.24)	0.45
	Non- current smokers	8,154	875	0.88 (0.81 to 0.96)		0.86 (0.80 to 0.94)		0.90 (0.82 to 0.97)		0.91 (0.85 to 0.99)	

B) Cardiovascular diseases

Subgroups		N	CVD events	Baseline PAEE HR (95%CI)	P†	ΔPAEE HR (95%CI)	P†	Baseline MDS HR (95%CI)	P†	ΔMDS HR (95%CI)	P†
All participants		8,800	2,540	0.93 (0.88 to 0.98)		0.94 (0.89 to 0.99)		0.94 (0.90 to 0.99)		0.93 (0.88 to 0.97)	
Age	<65 years	6,655	1,508	0.92 (0.87 to 0.98)	0.64	0.94 (0.89 to 1.00)	0.85	0.93 (0.88 to 0.99)	0.42	0.93 (0.87 to 0.98)	0.67
	≥65 years	2,145	1,032	0.93 (0.86 to 1.01)		0.94 (0.86 to 1.02)		0.97 (0.89 to 1.04)		0.93 (0.86 to 1.00)	
Sex	Male	3,527	1,238	0.92 (0.86 to 0.99)	0.88	0.94 (0.88 to 1.01)	0.54	0.95 (0.89 to 1.02)	0.54	0.94 (0.88 to 1.00)	0.59
	Female	5,273	1,302	0.94 (0.88 to 1.02)		0.94 (0.88 to 1.01)		0.93 (0.87 to 1.00)		0.91 (0.86 to 0.98)	
BMI	<25 kg/m²	3,898	965	0.89 (0.82 to 0.97)	0.11	0.92 (0.84 to 0.99)	0.24	0.90 (0.84 to 0.98)	0.25	0.96 (0.89 to 1.03)	0.19
	≥25 kg/m²	4,902	1,575	0.96 (0.90 to 1.02)		0.96 (0.90 to 1.02)		0.96 (0.90 to 1.02)		0.90 (0.85 to 0.96)	
Smoking	Current smokers	688	215	0.98 (0.82 to 1.17)	0.22	0.93 (0.78 to 1.12)	0.89	1.05 (0.89 to 1.25)	0.72	1.06 (0.91 to 1.23)	0.23
	Non- current smokers	8,112	2,325	0.92 (0.88 to 0.97)		0.94 (0.89 to 0.99)		0.92 (0.88 to 0.97)		0.92 (0.88 to 0.97)	

Figure S3 - Associations of mutually adjusted baseline and within-person changes in PAEE and MDS with A) incident DM; and B) incident CVD in different strata of baseline age, sex, BMI, and smoking status per 1-SD difference in each exposure, in the EPIC-Norfolk Study.

Analyses are based on Model 2 (see methods).

MDS=Mediterranean diet score; PAEE=physical activity energy expenditure

1-SD increment in baseline PAEE equals to 4.64 kJ/kg/day, in ΔPAEE equals to 0.65 kJ/kg/day per year, in baseline MDS equals to 1.30 points, and in ΔMDS equals to 0.33 points per year

†P values for interaction in subgroups.

Model 3

MDS (1)	PAEE (1)	MDS (2)	PAEE (2)	Trajectory group	N	Person years	DM events	A Hazard Ratio (95% CI); across all trajectories	B Hazard Ratio (95% CI); baseline stratified
L	L	L	L	G1	890	10,964	127	1.00 (ref)	1.00 (ref)
L	L	L	H	G2	268	3,728	24	0.68 (0.44 to 1.06)	0.68 (0.43 to 1.07)
L	L	H	L	G3	441	5,913	50	0.83 (0.60 to 1.16)	0.85 (0.60 to 1.20)
L	L	H	H	G4	146	2,216	10	0.55 (0.29 to 1.04)	0.54 (0.28 to 1.04)
L	H	L	L	G5	622	7,875	96	1.14 (0.87 to 1.49)	1.29 (0.97 to 1.71)
L	H	L	H	G6	1062	15,357	120	0.86 (0.67 to 1.12)	1.00 (ref)
L	H	H	L	G7	321	4,400	40	0.92 (0.64 to 1.32)	0.94 (0.64 to 1.37)
L	H	H	H	G8	512	7,571	47	0.84 (0.59 to 0.18)	0.90 (0.63 to 1.28)
H	L	L	L	G9	358	4,583	40	0.79 (0.55 to 1.13)	0.94 (0.64 to 1.36)
H	L	L	H	G10	121	1,707	14	0.87 (0.50 to 1.52)	1.03 (0.58 to 1.83)
H	L	H	L	G11	1138	15,607	123	0.83 (0.65 to 1.08)	1.00 (ref)
H	L	H	H	G12	386	5,596	41	0.93 (0.65 to 1.33)	1.19 (0.82 to 1.71)
H	H	L	L	G13	220	2,747	26	0.84 (0.55 to 1.28)	1.14 (0.72 to 1.81)
H	H	L	H	G14	381	5,312	50	1.06 (0.76 to 1.47)	1.54 (1.08 to 2.19)
H	H	H	L	G15	702	10,014	62	0.69 (0.69 to 0.93)	0.94 (0.67 to 1.31)
H	H	H	H	G16	1286	19,438	98	0.74 (0.56 to 0.97)	1.00 (ref)

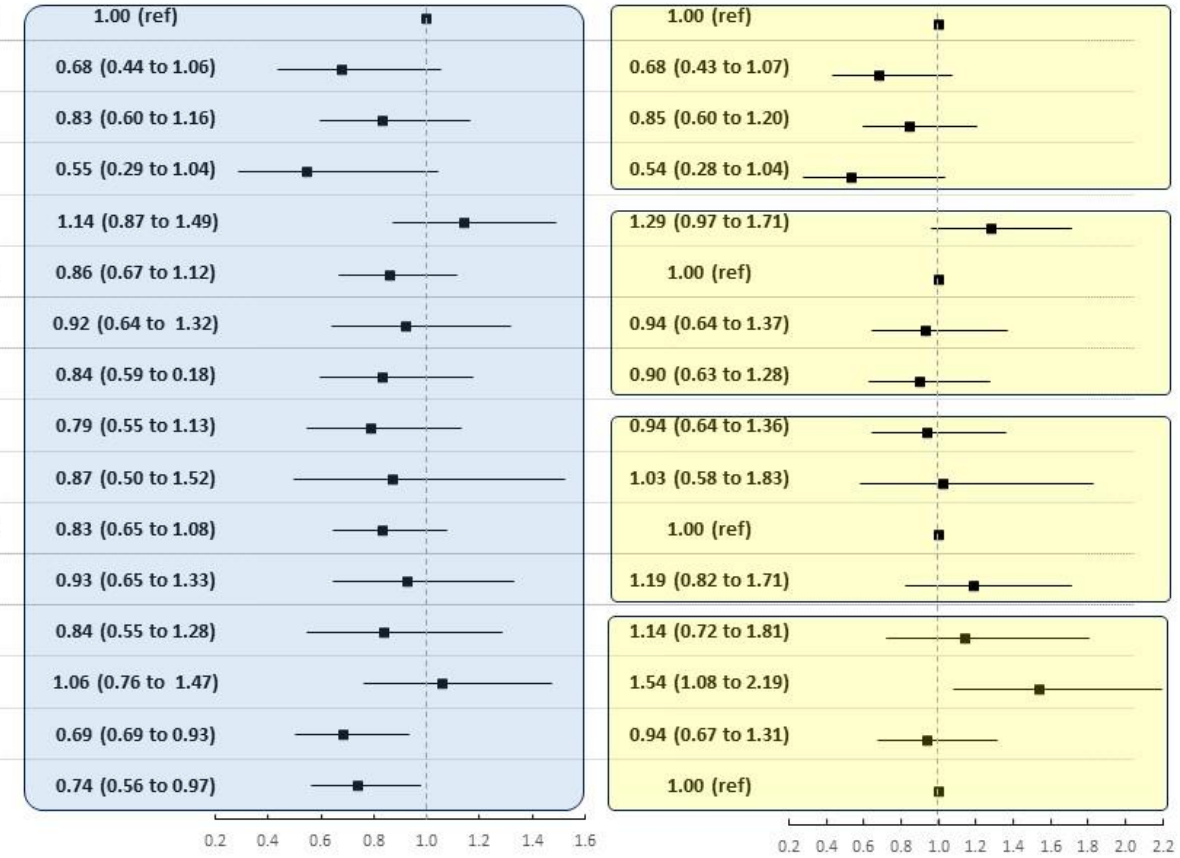

Figure S4 -Joint associations of different trajectories of physical activity and diet quality with incident DM in the EPIC-Norfolk Study; A) comparison across all trajectories together (G1 as reference); B) stratified by baseline exposure level (stable behaviours as references).

MDS (1) =Mediterranean diet score at baseline assessment; PAEE (1)=physical activity energy expenditure at baseline assessment, MDS (2) = MDS at repeated assessment; PAEE (2)=PAEE at repeated assessment; L=Low; H=High.

Analyses are based on Model 3 (see methods). High diet quality cut-off: MDS ≥ 8.5 points; high PA cut-off: PAEE ≥ 5 kJ/kg/day.

Model 3

MDS (1)	PAEE (1)	MDS (2)	PAEE (2)	Trajectory group	N	Person years	CVD events	A Hazard Ratio (95% CI); across all trajectories	B Hazard Ratio (95% CI); baseline stratified
L	L	L	L	G1	891	11,724	321	1.00 (ref)	1.00 (ref)
L	L	L	H	G2	267	3,857	83	1.13 (0.89 to 1.44)	1.13 (0.88 to 1.45)
L	L	H	L	G3	426	5,977	140	0.96 (0.79 to 1.18)	0.92 (0.74 to 1.13)
L	L	H	H	G4	143	2,258	37	0.85 (0.60 to 1.19)	0.81 (0.57 to 1.14)
L	H	L	L	G5	626	8,027	236	1.18 (0.99 to 1.39)	1.25 (1.04 to 1.50)
L	H	L	H	G6	1,048	16,196	272	0.93 (0.79 to 1.10)	1.00 (ref)
L	H	H	L	G7	312	4,521	91	0.83 (0.65 to 1.05)	0.90 (0.70 to 1.15)
L	H	H	H	G8	505	8,094	125	0.94 (0.76 to 1.16)	1.01 (0.81 to 1.26)
H	L	L	L	G9	354	4,669	137	1.11 (0.91 to 1.36)	1.18 (0.95 to 1.45)
H	L	L	H	G10	124	1,850	37	1.10 (0.78 to 1.55)	1.18 (0.83 to 1.68)
H	L	H	L	G11	1,136	16,503	333	0.93 (0.80 to 1.09)	1.00 (ref)
H	L	H	H	G12	383	5,924	103	1.00 (0.80 to 1.26)	1.07 (0.85 to 1.34)
H	H	L	L	G13	221	2,829	85	1.16 (0.91 to 1.47)	1.45 (1.11 to 1.88)
H	H	L	H	G14	381	5,886	89	0.83 (0.65 to 1.05)	1.08 (0.84 to 1.39)
H	H	H	L	G15	687	10,046	204	0.92 (0.77 to 1.10)	1.16 (0.96 to 1.41)
H	H	H	H	G16	1,296	21,121	247	0.78 (0.66 to 0.93)	1.00 (ref)

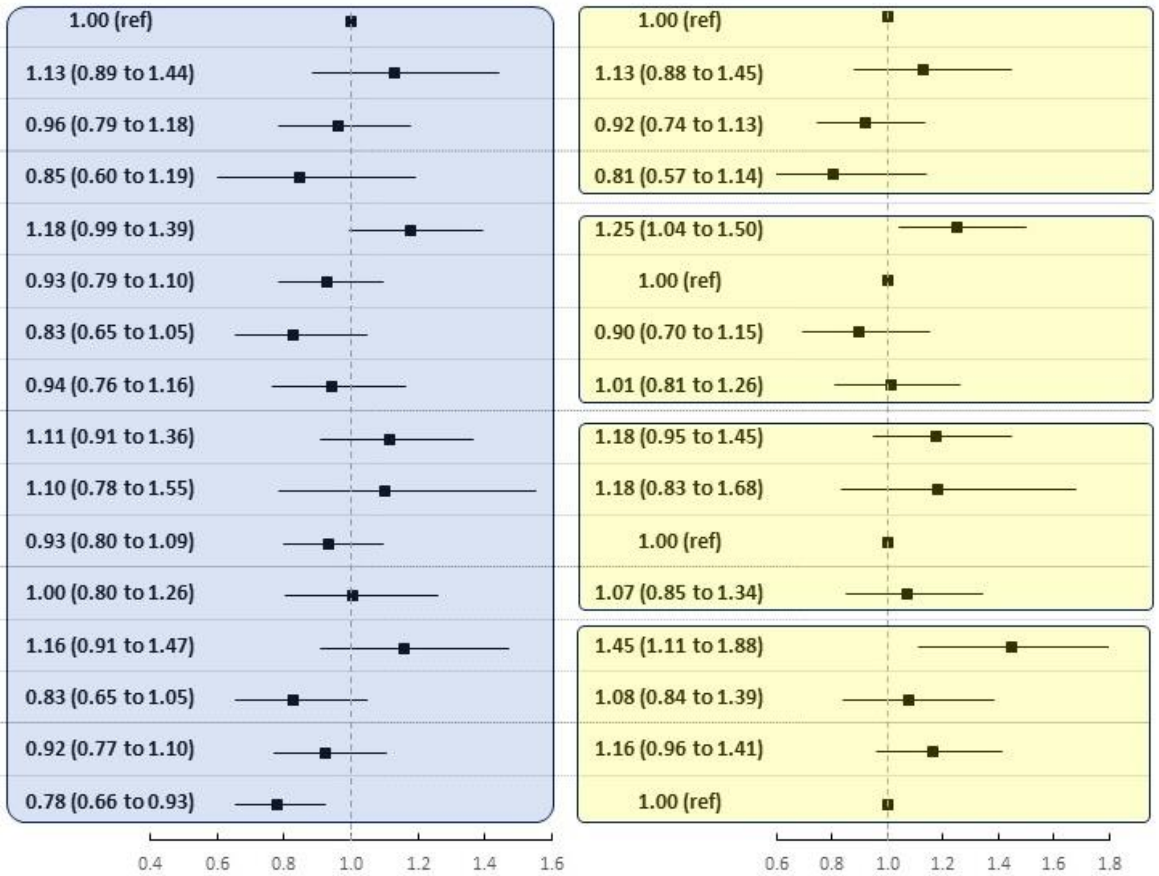

Figure S5 -Joint associations of different trajectories of physical activity and diet quality with incident CVD in the EPIC-Norfolk Study; A) comparison across all trajectories together (G1 as reference); B) stratified by baseline exposure level (stable behaviours as references).

MDS (1) =Mediterranean diet score at baseline assessment; PAEE (1)=physical activity energy expenditure at baseline assessment, MDS (2) = MDS at repeated assessment; PAEE (2)=PAEE at repeated assessment; L=Low; H=High.
Analyses are based on Model 3 (see methods). High diet quality cut-off: MDS ≥ 8.5 points; high PA cut-off: PAEE ≥ 5 kJ/kg/day.

A

Incidence of DM

Repeated PAEE level \ Baseline PAEE level	Low 0	Medium 5.3 (1.7)	High 11.6 (2.7)
High 11.7 (2.7)	0.71 (0.39 to 1.31)	0.64 (0.47 to 0.86)	0.62 (0.48 to 0.80)
Medium 5.2 (1.7)	0.77 (0.57 to 1.02)	0.81 (0.66 to 0.99)	0.91 (0.72 to 1.15)
Low 0	1.00 (ref)	0.86 (0.69 to 1.08)	0.97 (0.70 to 1.34)

Incidence of CVD

Repeated PAEE level \ Baseline PAEE level	Low 0	Medium 5.3 (1.7)	High 11.6 (2.7)
High 11.6 (2.7)	1.25 (0.89 to 1.75)	0.72 (0.60 to 0.87)	0.79 (0.68 to 0.93)
Medium 5.2 (1.7)	0.96 (0.81 to 1.14)	0.83 (0.73 to 0.94)	0.80 (0.69 to 0.93)
Low 0	1.00 (ref)	1.01 (0.88 to 1.14)	1.16 (0.95 to 1.41)

B

Incidence of DM

Repeated MDS level \ Baseline MDS level	Low 7.1 (0.7)	Medium 8.6 (0.3)	High 10.0 (0.7)
High 10.1 (0.6)	0.72 (0.48 to 1.08)	0.70 (0.54 to 0.92)	0.78 (0.63 to 0.96)
Medium 8.7 (0.3)	0.91 (0.72 to 1.15)	0.85 (0.68 to 1.06)	0.73 (0.56 to 0.94)
Low 7.3 (0.7)	1.00 (ref)	0.86 (.68 to 1.09)	1.31 (0.94 to 1.83)

Incidence of CVD

Repeated MDS level \ Baseline MDS level	Low 7.1 (0.7)	Medium 8.6 (0.3)	High 10.0 (0.7)
High 10.1 (0.6)	0.79 (0.61 to 1.01)	0.89 (0.76 to 1.04)	0.87 (0.76 to 0.99)
Medium 8.7 (0.3)	0.94 (0.81 to 1.09)	0.84 (0.73 to 0.97)	1.00 (0.85 to 1.16)
Low 7.3 (0.7)	1.00 (ref)	0.99 (0.85 to 1.14)	1.19 (0.96 to 1.47)

C

Incidence of DM

ΔMDS \ ΔPAEE	Decreasers ≤ -0.1	Maintainers -0.1 to 0.1	Increasers ≥ 0.1
Increasers ≥ 0.1	0.84 (0.66 to 1.06)	0.80 (0.62 to 1.04)	0.66 (0.49 to 0.89)
Maintainers 0.1 to 0.1	0.96 (0.75 to 1.25)	0.94 (0.72 to 1.24)	0.77 (0.56 to 1.07)
Decreasers ≤ -0.1	1.00 (ref)	0.82 (0.64 to 1.07)	0.68 (0.50 to 0.93)

Incidence of CVD

ΔMDS \ ΔPAEE	Decreasers ≤ -0.1	Maintainers -0.1 to 0.1	Increasers ≥ 0.1
Increasers ≥ 0.1	0.88 (0.76 to 1.02)	0.83 (0.71 to 0.98)	0.75 (0.62 to 0.90)
Maintainers 0.1 to 0.1	1.06 (0.91 to 1.25)	0.87 (0.74 to 1.04)	0.84 (0.69 to 1.03)
Decreasers ≤ -0.1	1.00 (ref)	0.90 (0.77 to 1.05)	0.92 (0.76 to 1.10)

Figure S6 - Associations of different trajectories of PA and diet with incidence of DM and CVD in the EPIC-Norfolk Study, based on three-by-three levels of exposures

Analyses are based on Model 2 (see methods). MDS=Mediterranean diet score; PAEE=physical activity energy expenditure; ΔMDS=over time changes in MDS; ΔPAEE=over time changes in PAEE.
A) Model mutually adjusted for baseline and repeated MDS. Cut offs for PAEE: Low PAEE = no PA reported, Medium PAEE = below 10 kJ/kg/day (WHO recommendations for additional health benefits), High PAEE = equal or above 10 kJ/kg/day.
B) Model mutually adjusted for baseline and repeated PAEE. Cut offs for MDS: Low MDS = 1st Tertile of MDS in population (< 8 points), Medium MDS 2nd Tertile of MDS in population (≥ 8 and ≤ 9.1 points), High MDS = 3rd Tertile of MDS in population (> 9.1 points).
C) Model mutually adjusted for baseline PAEE and baseline MDS. ΔPAEE is expressed in kJ/kg/day per year, ΔMDS is expressed in points per year.

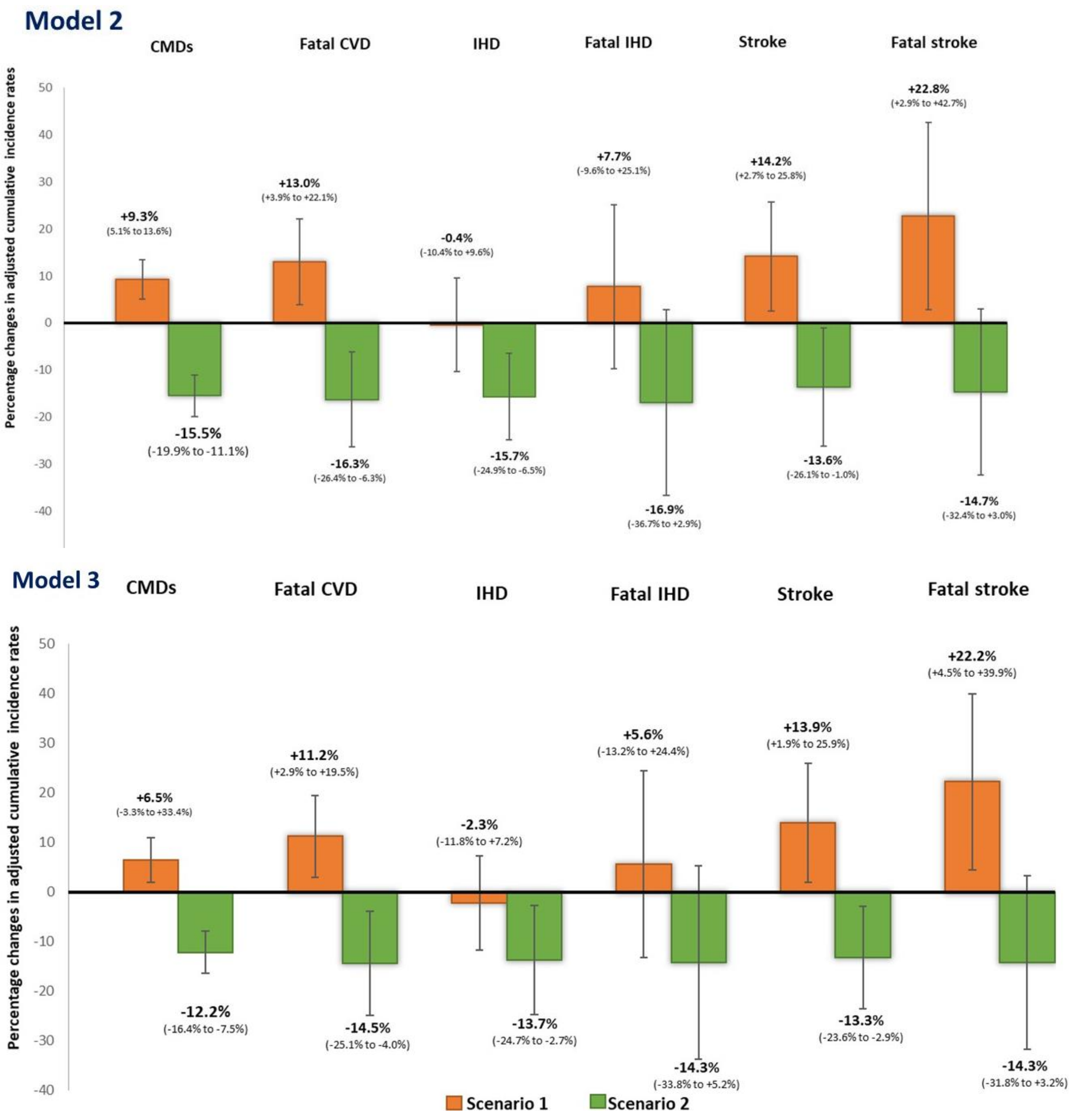

Figure S7 - Population impact by estimating changes in cumulative adjusted incident rates of any CMD (DM or CVD) and subtypes of CVD in the population during two decades of follow-up, under two counterfactual scenarios.

Counterfactual scenario 1; applying the adjusted incident rates of trajectory group 1 (G1, low PA and low diet quality at both assessments) to the whole population.
Counterfactual scenario 2; applying the adjusted incident rates of trajectory group 16 (G16, high PA and high diet quality at both assessments) to the whole population. See Figure 2 and the methods section for the definition of each trajectory group.
Error bars indicate 95% confidence intervals (95% CI) for percentage of changes in adjusted incident rate of the population under counterfactual scenarios, compared with adjusted incident rate under observed physical activity and diet exposures.
Analyses are based on Model 2 and Model 3 (see methods).

**Table S8-** Population impact by estimating the differences in total number of DM cases that could have been potentially observed under two different counterfactual scenarios.									
**Trajectory Group (% of total sample)†**	**Scenario under observed exposures**			**Counterfactual scenario 1** Applying the adjusted mortality rate of G1 to all groups			**Counterfactual scenario 2** Applying the adjusted mortality rate of G16 to all groups		
	Crude cases	Adjusted incident rate‡	Adjusted number of cases	Number of cases expected	Difference from the number of cases observed	Percentage of contribution to total difference	Number of cases expected	Difference from the number of cases observed	Percentage of contribution to total difference
G1 (10.1%)	127	1158	144	144	Ref	Ref	91	-53	20.7
G2 (3.0%)	24	644	29	44	14	5.2	28	-2	0.7
G3 (5.0%)	50	846	57	71	15	5.3	45	-12	4.6
G4 (1.6%)	10	451	12	24	12	4.3	15	3	-1.2
G5 (7.0%)	96	1219	108	102	-7	-2.4	64	-44	17.1
G6 (12.0%)	120	781	140	173	33	12.2	109	-31	11.9
G7 (3.6%)	40	909	45	53	7	2.6	33	-12	4.8
G8 (5.8%)	47	621	60	83	24	8.7	53	-7	2.7
G9 (4.0%)	40	873	48	58	10	3.6	37	-12	4.5
G10 (1.4%)	14	820	17	19	2	0.8	12	-5	2.0
G11 (12.9%)	123	788	152	184	32	11.6	116	-36	14.0
G12 (4.4%)	41	733	52	62	11	3.9	39	-12	4.8
G13 (2.5%)	26	946	33	36	3	0.9	23	-11	4.2
G14 (4.3%)	50	941	60	62	2	0.9	39	-20	7.9
G15 (7.9%)	62	619	76	115	39	14.2	72	-3	1.3
G16 (14.5%)	98	504	132	208	77	28.1	132	Ref	Ref
Sum	968		1165	1438	+273 (Standard Error: 115)§	100%	908	-257 (Standard Error: 106)§	100%
Percentage of difference in total incident diseases of counterfactual scenarios vs. observed adjusted deaths (95% CI)				+23.6% (%4.1 to 42.9%)§			-22.0% (-39.9% to -4.1%)§		

†G = group. See figure 1 for the definition of each trajectory group.

‡Adjusted incident rate is expressed as cases per 10^5 person-years and is based on covariates in the Model 2 (see methods).

§ Standard error of the count or percent points, estimated with a bootstrap technique.

Table S9- Population impact by estimating the differences in total number of CVD cases that could have been potentially observed under two different counterfactual scenarios.

Trajectory Group (% of total sample)[†]	Scenario under observed exposures			Counterfactual scenario 1 Applying the adjusted mortality rate of G1 to all groups			Counterfactual scenario 2 Applying the adjusted mortality rate of G16 to all groups		
	Crude cases	Adjusted incident rate[‡]	Adjusted number of cases	Number of cases expected	Difference from the number of cases observed	Percentage of contribution to total difference	Number of cases expected	Difference from the number of cases observed	Percentage of contribution to total difference
G1 (10.1%)	321	2738	333	333	Ref	Ref	268	-65	13.1
G2 (3.0%)	83	2152	108	100	-8	-5.5	81	-28	5.6
G3 (4.8%)	140	2342	155	159	5	3.1	128	-27	5.3
G4 (1.6%)	37	1638	47	53	6	4.2	43	-4	0.9
G5 (7.1%)	236	2940	267	236	-31	-21.1	190	-77	15.5
G6 (11.9%)	272	1679	372	394	22	14.9	317	-55	11.1
G7 (3.5%)	91	2013	104	118	14	9.4	95	-9	1.8
G8 (5.7%)	125	1544	182	190	7	4.9	153	-30	6.0
G9 (4.0%)	137	2934	144	132	-12	-8.0	106	-38	7.6
G10 (1.4%)	37	2000	49	46	-3	-2.0	37	-12	2.4
G11 (12.9%)	333	2018	398	423	26	17.5	340	-57	11.5
G12 (4.4%)	103	1739	139	143	4	2.5	115	-24	4.9
G13 (2.5%)	85	3005	94	83	-10	-7.1	67	-27	5.4
G14 (4.3%)	89	1512	123	143	20	13.5	115	-8	1.6
G15 (7.8%)	204	2031	245	258	14	9.2	208	-37	7.4
G16 (14.7%)	247	1169	390	485	95	64.5	390	Ref	Ref
Sum	2540		3150	3297	+147 (Standard Error: 79)§	100%	2652	-498 (Standard Error: 96)§	100%
Percentage of difference in total incident diseases of counterfactual scenarios vs. observed adjusted deaths (95% CI)				+4.7% (-0.2% to +9.6%)§			-15.8% (-21.8% to -9.4%)§		

†G = group. See figure 2 for the definition of each trajectory group.

‡ Adjusted incident rate is expressed as cases per 10^5 person-years and is based on covariates in the Model 2 (see methods).

§ Standard error of the count or percent points, estimated with a bootstrap technique.
